# Regioselective construction of two isomeric BN-fused aromatic frameworks enabling the synthesis of ultralong room-temperature phosphorescence materials

**DOI:** 10.1039/d5sc05061h

**Published:** 2025-08-25

**Authors:** Qiang Feng, Junxiong Yao, Qianxin Wu, Yang Qiu, Zicheng Wang, Xia Wang, Weilin Chen, Sibo Tong, Xiaohua Cao, Jianqi Sun, Qianqian Ye, Jianhua Liu, Dianyuan Wang, Jianguo Wang, Huanan Huang

**Affiliations:** a College of Chemistry and Chemical Engineering, Jiangxi Province Engineering Research Center of Ecological Chemical Industry, Jiujiang University Jiujiang 332005 China huanan200890@163.com; b College of Chemistry and Chemical Engineering, College of Green Chemistry and Environment, Institutes of Biomedical Sciences, Inner Mongolia Key Laboratory of Synthesis and Application of Organic Functional Molecules, Inner Mongolia University Hohhot 010021 P. R. China wangjg@iccas.ac.cn

## Abstract

BN-fused aromatic compounds have garnered significant attention due to their unique electronic structures and exceptional photophysical properties, positioning them as highly promising candidates for applications in organic optoelectronics. However, the regioselective synthesis of BN isomers remains a formidable challenge, primarily stemming from the difficulty in precisely controlling reaction sites, limiting structural diversity and property tunability. Herein, we propose a regioselective synthetic strategy that employs 2,1-BN-naphthalene derivatives, wherein selective activation of N–H and C–H bonds is achieved in conjunction with *ortho*-halogenated phenylboronic acids. Under uniform reaction conditions, two distinct boron–nitrogen fused ring isomers were successfully synthesized. A computational analysis of C–X bond dissociation energies indicates that the observed regioselectivity is most likely governed by the interplay of halogen electronegativity, atomic radius, and bond dissociation energy parameters. Interestingly, the two isomers exhibit markedly distinct room-temperature phosphorescence (RTP) in polyvinyl alcohol (PVA). Specifically, 3a@PVA demonstrates ultralong RTP characteristics, featuring an exceptionally long phosphorescence lifetime of up to 2388.2 ms and an afterglow persisting for more than 30 seconds, significantly longer than the 286.1 ms observed for 4a@PVA. Theoretical investigations reveal that 3a possesses a higher spin–orbit coupling constant and more intersystem crossing channels than 4a. Additionally, the dual-sided fixation of the 3a@PVA system imposes significant constraints on intramolecular motions, effectively suppressing non-radiative decay pathways, which accounts for its distinct afterglow behaviors. These divergent photophysical characteristics significantly enhance their potential applications in advanced anti-counterfeiting technologies. This work not only establishes a versatile synthetic strategy for the regioselective construction of BN-fused isomers but also provides fundamental insights into the rational design of BN-incorporating organic RTP systems.

## Abstract 2

A halogen-regulated strategy enabled regioselective synthesis of BN aromatics. Embedding them in a PVA matrix yielded RTP materials with a 2388.2 ms lifetime and 30 s afterglow, offering potential for applications and guidance for molecular design.

## Graphical abstract

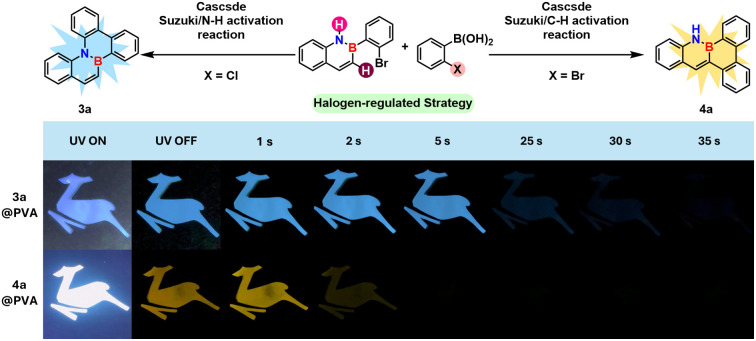

## Introduction

**Fig. 1:**
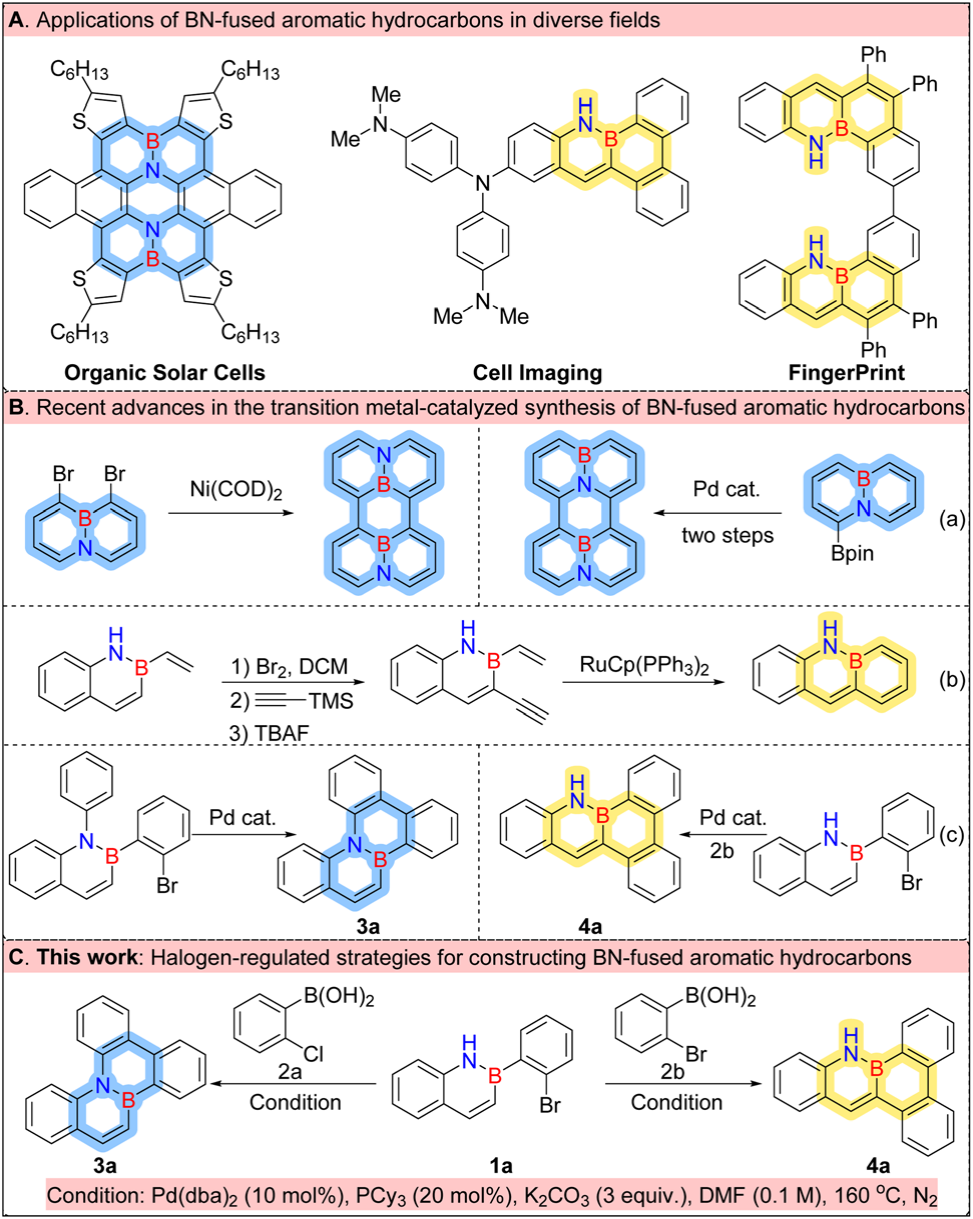
(A) Applications of BN-fused aromatic hydrocarbons in diverse fields. (B) Transition metal-catalyzed synthesis of BN-fused aromatic compounds. (C) Halogen-regulated strategies for constructing BN-fused aromatic hydrocarbons.

Boron–nitrogen (BN) aromatic compounds have garnered significant attention in recent years owing to their distinctive physicochemical properties and have broad applications in organic synthesis, materials science, and related interdisciplinary fields.^1–5^ In contrast to conventional all-carbon aromatics, BN aromatics feature isoelectronic substitution of C=C units with B–N units, which perturbs the π-conjugated system and endows the molecules with distinct electronic characteristics, enhanced thermal stability, and tuneable optical properties.^6–8^ These features make BN aromatics highly promising for applications in optoelectronic devices,^9–13^ chemical sensing,^14–20^ and pharmaceutical design^21–24^ (Fig. 1A). Despite their potential, synthesizing BN aromatics remains a formidable challenge. Traditional approaches often involve electrophilic borylation under harsh conditions or multistep protocols, which suffer from low efficiency, poor atom economy, and limited structural diversity.^25,26^ Consequently, the development of efficient, selective, and broadly applicable synthetic strategies is essential not only for advancing BN chemistry but also for realizing their potential in diverse application domains.

Recent advances in transition metal-catalysed coupling reactions and C–H activation strategies have significantly expanded the range of tools available for constructing boron–nitrogen (BN)-fused polycyclic frameworks. Notably, Cui and his colleagues pioneered a tandem insertion/C–H activation strategy for synthesising boron–nitrogen (BN)-embedded tetraphene derivatives.^27^ In parallel, palladium-catalysed methodologies reported by Liu^28^ and Park^29^ have facilitated the efficient assembly of diverse BN aromatic scaffolds. Nickel-catalysed systems developed by Wang^7^ and Song^30^ have also demonstrated excellent reactivity and functional group tolerance (Fig. 1B(a)). Furthermore, catalytic systems based on rhodium,^31–33^ ruthenium,^34,35^ and gold^36–38^ have shown promising potential in related transformations (Fig. 1B(b)). Our group is dedicated to constructing boron–nitrogen (BN) aromatic frameworks. We have developed an efficient C–H activation protocol utilising diverse substrates and optimised reaction conditions to successfully synthesise compounds 3a (ref. 17) and 4a (ref. 39) (Fig. 1B(c)). However, this strategy imposes stringent requirements on the substrate structure. Despite these achievements, challenges remain, particularly with regard to site selectivity, which is often compromised by the intrinsic electronic and steric properties of the substrates. Therefore, integrating transition metal catalysis with rational substrate design to achieve regioselective functionalisation is a key way of streamlining the synthesis of structurally diverse BN-containing aromatics.

Herein, we present a halogen-regulated synthetic approach that exploits the differential reactivity patterns of aryl halides to govern the sequential order of bond-forming processes. Compound 1a was selected as the precursor core, featuring two distinct types of reactive hydrogen sites-C(3)–H and N–H. The reactivity hierarchy of these sites is governed by the intrinsic properties of the aryl halide substrate, thereby facilitating selective pathway modulation. As anticipated, this synthetic strategy successfully afforded a pair of BN-fused aromatic isomers, namely compounds 3 and 4. Moreover, photophysical analyses demonstrated that both compounds exhibit RTP phenomena when incorporated into a PVA matrix. However, the two isomers embedded in PVA exhibit marked differences in RTP lifetime and emission color. Notably, the 3a@PVA system exhibits an exceptionally long phosphorescence lifetime of 2388.2 ms and visible afterglow persistence for up to over 30 seconds under ambient conditions. Additionally, patterned emissive images with well-defined motifs were successfully obtained, underscoring the potential of this system for applications in anti-counterfeiting and information display technologies. This work provides a novel synthetic approach for designing BN-fused aromatic compounds with excellent properties, providing a valuable opportunity to explore the relationship between the structure and RTP properties, which is of considerable importance for advancing the development of boron–nitrogen functional molecules with enhanced performance.

## Results and discussion

Our investigation initially centered on the coupling reaction between 2,1-BN naphthalene (1a) and *ortho*-chlorophenylboronic acid (2a), under a variety of conditions employing different palladium catalysts, phosphine ligands, and bases (Table 1). The reaction was first conducted in *N*,*N*-dimethylformamide (DMF) at 160 °C using 10 mol% Pd(OAc)_2_, 20 mol% PCy_3_, and 3 equivalents of Na_2_CO_3_ over 24 hours, affording the desired product 3a in 18% isolated yield (Table 1, entry 1). Encouraged by this initial result, we performed a systematic optimization of the catalytic system. Screening of palladium sources revealed that Pd(dba)_2_ significantly improved the yield to 54% (entry 2), outperforming other tested catalysts such as Pd(PPh_3_)_2_Cl_2_ and Pd(dppf)Cl_2_. Subsequent ligand screening included sterically demanding X-phos (entry 5), less hindered PPh_3_ (entry 6), and bidentate dppf (entry 7), none of which led to further yield enhancement. Evaluation of bases revealed that replacing Na_2_CO_3_ with K_2_CO_3_ markedly improved the yield of 3a to 85% (entry 8), while weaker bases such as NaHCO_3_ gave lower yields (63%, entry 9). Stronger bases like Cs_2_CO_3_ and NaOAc resulted in diminished efficiency, attributable to decomposition of the sensitive BN substrate under overly basic conditions (entries 10 and 11). To further explore the halogen-regulated pathway, 2-chlorophenylboronic acid (2a) was replaced with its bromo analogue 2b under the optimized reaction conditions. Interestingly, this modification led to the formation of a distinct BN-fused product, 4a, in 53% isolated yield, thus demonstrating the impact of halogen substitution on the reaction trajectory and product outcome.

**Table 1:** Optimization conditions^a^

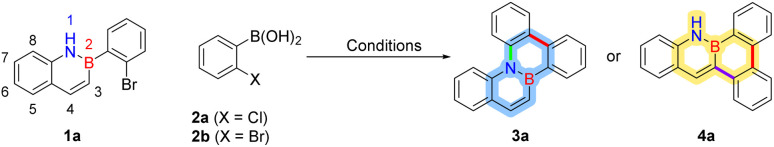				
Entries	Cat.	Ligand	Base	Yield^b^
1	Pd(OAc)_2_	PCy_3_	Na_2_CO_3_	18%
2	Pd(dba)_2_	PCy_3_	Na_2_CO_3_	54%
3	Pd(PPh_3_)Cl_2_	PCy_3_	Na_2_CO_3_	13%
4	Pd(dppf)Cl_2_	PCy_3_	Na_2_CO_3_	15%
5	Pd(dba)_2_	X-phos	Na_2_CO_3_	48%
6	Pd(dba)_2_	PPh_3_	Na_2_CO_3_	68%
7	Pd(dba)_2_	dppf	Na_2_CO_3_	46%
8	Pd(dba)_2_	PCy_3_	K_2_CO_3_	85%
9	Pd(dba)_2_	PCy_3_	NaHCO_3_	63%
10	Pd(dba)_2_	PCy_3_	Cs_2_CO_3_	14%
11	Pd(dba)_2_	PCy_3_	NaOAc	23%
12^c^	Pd(dba)_2_	PCy_3_	K_2_CO_3_	53%

a Reaction conditions: 1a (1.0 equiv., 0.2 mmol), 2a (1.0 equiv., 0.2 mmol), Pd-cat. (10 mol%), ligand (20 mol%), base (3.0 equiv., 0.6 mmol), DMF (1.0 mL), 160 °C, 24 h, N_2_ atmosphere.

b Isolated yield.

c 1a (1.0 equiv., 0.2 mmol) and 2b (1.0 equiv., 0.2 mmol) as substrates.

Under the optimized conditions, we successfully synthesized a series of BN-fused aromatic compounds, as summarized in Table 2. The coupling of various *ortho*-chlorophenylboronic acids with 1a proceeded smoothly, affording products 3a, 3b, and 3d in moderate to good yields. However, when an electron-deficient arylboronic acid was employed, the yield of 3c dropped significantly to 23%, likely as a result of the diminished nucleophilicity of the boronic acid.^40^ Notably, substitution of compound 1 with electron-withdrawing fluorine atoms had little effect on the reaction efficiency: target compounds 3e and 3f were obtained in satisfactory yields, highlighting the robustness of the protocol toward electronic variations in the BN framework. We next turned our attention to the reactivity of *ortho*-bromophenylboronic acid under the same conditions. The corresponding coupling with 1a afforded product 4a in 53% yield, confirming the viability of the C–H activation pathway. In contrast, employing thiophene boronic acid as the coupling partner resulted in a reduced yield of 15% for 4b, likely due to the strong coordination of the sulfur heteroatom with the catalyst, which hampers C–H activation efficiency and promotes competing side reactions in the electron-deficient heterocyclic scaffold.^41^ Similarly, the coupling of an electron-deficient variant of 1a afforded product 4c in only 30% yield, likely attributable to diminished reactivity at the C(3) position of the electron-poor BN scaffold, arising from the electron-withdrawing group reducing the arene's electron density and thereby impeding catalyst coordination and C–H activation efficiency.^42^ These results underscore the critical influence of electronic effects on both boronic acid and BN substrate reactivity in these halogen-regulated transformations.

**Table 2:** Substrate scope^a^

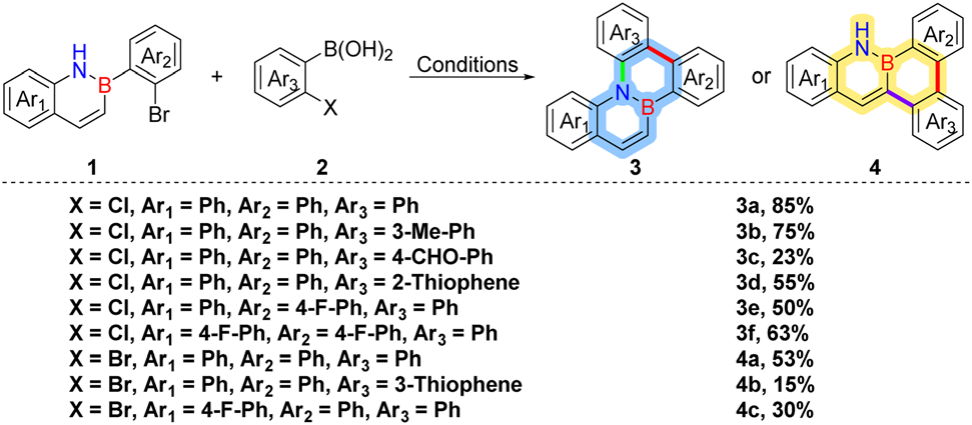

a Conditions: 1 (1.0 equiv., 0.2 mmol), 2 (1.0 equiv., 0.2 mmol), Pd(dba)_2_ (10 mol%), PCy_3_ (20 mol%), K_2_CO_3_ (3 equiv., 0.6 mmol), DMF (1 mL), 160 °C, 12 h, N_2_ atmosphere.

**Fig. 2:**
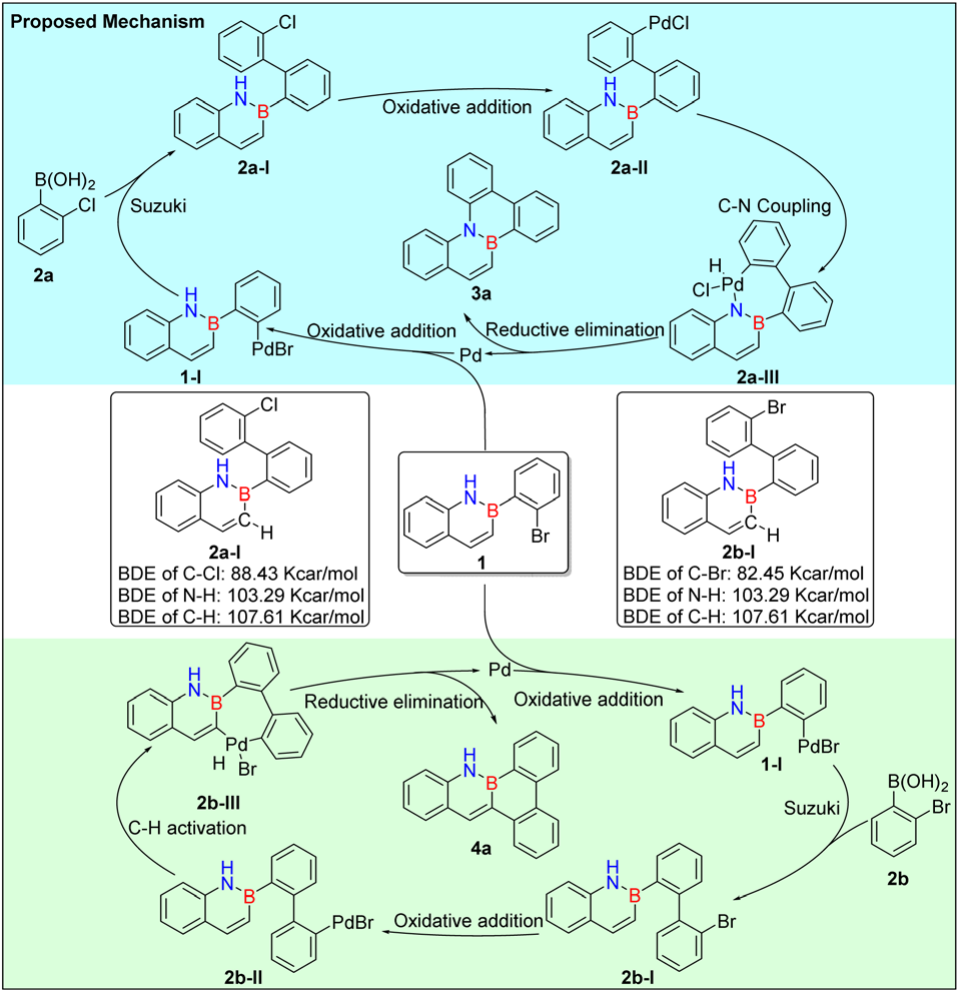
Proposed mechanism for the synthesis of compounds 3a and 4a.

Based on our experimental observations, we propose a plausible reaction mechanism for this halogen-regulated transformation (Fig. 2). The initial step involves a Suzuki–Miyaura coupling of 1a with either 2a or 2b, affording intermediates 2a-I and 2b-I, respectively. The subsequent divergence in the reaction pathway is governed by the nature of the carbon–halogen bond. Intermediate 2a-I, bearing a less reactive C–Cl bond, preferentially undergoes Buchwald–Hartwig amination *via* N–H bond activation to deliver product 3a. In contrast, the more reactive C–Br bond in intermediate 2b-I facilitates a C–H activation pathway, resulting in the formation of 4a. This mechanistic rationale accounts for the observed regioselectivity and highlights a direct correlation between halogen identity and pathway preference. To gain deeper insight into the origin of this selectivity, we conducted a comparative analysis of the transition states associated with N–H *versus* C–H activation in both intermediates. For substrate 2a, several factors favor the N–H activation pathway: the high bond dissociation energy of the C–Cl bond (88 kJ mol^−1^), the strong electronegativity of chlorine, and the favorable coordination ability of the nitrogen atom collectively stabilize the transition state.^43^ Furthermore, the relatively low bond dissociation energy of the N–H bond (103 kJ mol^−1^) lowers the activation barrier, rendering N–H cleavage kinetically accessible. In contrast, for substrate 2b, the reaction is biased toward C–H activation. This preference is attributed to the lower bond dissociation energy of the C–Br bond (82.1 kJ mol^−1^) and the larger atomic radius and weaker electronegativity of bromine. These features reduce steric hindrance and enhance the electrophilicity of the adjacent C–H bond, thereby facilitating its activation by the transition metal catalyst. Collectively, these results underscore the critical role of halogen identity in dictating divergent bond activation pathways and product selectivity in BN-fused aromatic ring construction.

**Fig. 3:**
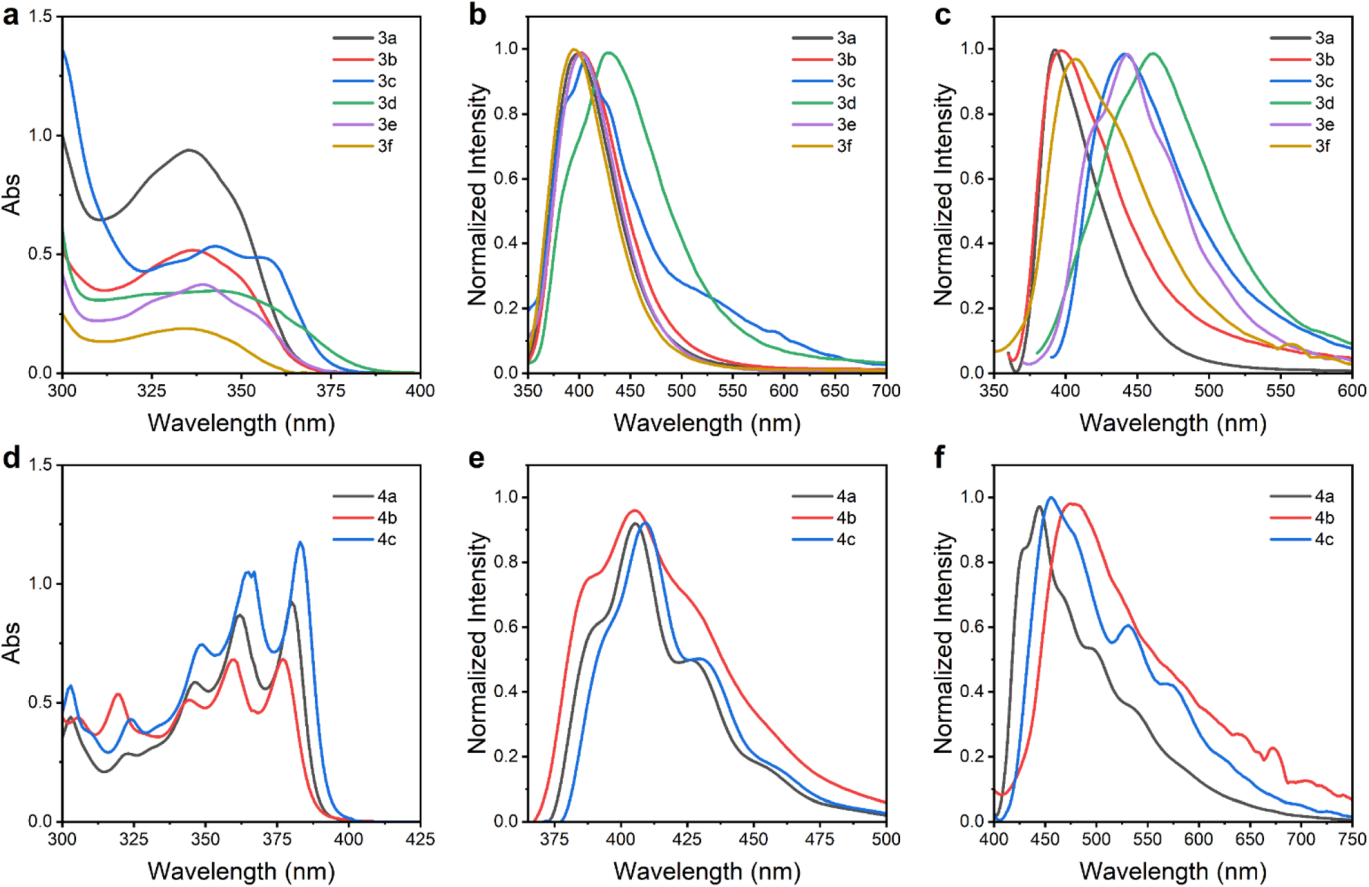
(a) The UV-vis spectra of 3a–3f in DCM (concentration = 10^−5^ M). (b) Fluorescence spectra of 3a–3f in DCM. (c) Solid-state fluorescence spectra of 3a–3f. (d) The UV-vis spectra of 4a–4c in DCM (concentration = 10^−5^ M). (e) Fluorescence spectra of 4a–4c in DCM. (f) Solid-state fluorescence spectra of 4a–4c.

Following the successful synthesis of BN-fused aromatic compounds, the UV-vis absorption, fluorescence emission and quantum yield of compounds 3 and 4 were systematically investigated (Fig. 3 and Table 3). Compounds 3a–3f exhibited absorption maxima in the range of 335–357 nm (Fig. 3a). Notably, compound 3c showed a significant redshift relative to 3a, which can be attributed to the enhanced π-conjugation induced by the aldehyde group (C=O). In contrast, compounds 4a–4c displayed multiple absorption bands, with intense peaks appearing between 376 and 383 nm (Fig. 3d)—significantly redshifted compared to their counterparts in series 3—highlighting the critical influence of the position of BN units within the molecular framework on the electronic structure and absorption characteristics. Photoluminescence studies revealed that the two series of isomers exhibit emissive behaviour in solution and the solid state. In dichloromethane, compounds 3a–3f displayed fluorescence emission bands ranging from 393 to 430 nm (Fig. 3b). Among them, 3d exhibited a pronounced redshift (*λ*_em_ = 430 nm), likely due to the introduction of a sulfur atom, which enhances π-conjugation—a phenomenon consistent with previously reported observations.^44,45^ However, compounds 4a–4c exhibited negligible red-shifted emission wavelengths, spanning the range of 404–409 nm (Fig. 3e). Notably, these compounds also exhibit solid-state fluorescence, with emission wavelengths red-shifted relative to their solution-state counterparts. In the case of 3-series compounds, incorporating sulfur atoms or aldehyde groups into the molecular framework results in a red shift in solid-state emission compared to 3a (Fig. 3c). Conversely, the 4-series compounds exhibit negligible variations in their solid-state fluorescence profiles. These findings highlight the potential of these compounds for applications in solid-state optoelectronic materials.

**Table 3:** Photophysical properties of 3a–3f and 4a–4c in dichloromethane and in the solid state

Compound	*λ*_max_, abs^a^ [nm]	*λ*_max_, em^a^ [nm]	Stokes shifts [cm^−1^] in DCM	*Φ*_F_ b [%]	*λ*_max_, em^c^ [nm]	Solid state Stokes shifts [cm^−1^]	*Φ*_F_ b ^,^ c [%]
3a	335	396	4598	0.19^d^	392	4340	3.3^d^
3b	337	402	4798	8.58	397	4485	4.65
3c	357	408	3501	0	441	5335	0.18
3d	347	430	5563	0	460	7079	0
3e	339	402	4623	7.50	442	6874	0.28
3f	335	394	4470	8.32	405	5159	0.22
4a	380	404	1563	69.80^d^	443	3741	49.30^d^
4b	376	404	1843	20.79	476	5587	0
4c	383	409	1660	67.37	455	4132	16.66

a All experiments were performed in DCM solution at 10^−5^ M.

b Absolute quantum yield determined using a calibrated integrating sphere system within ±3%; the absolute quantum yield was measured in DCM at room temperature.

c All experiments were performed in the solid state.

d The relevant data were reported previously.^17,39^

Furthermore, the photoluminescence quantum yields (*Φ*) were found to be highly sensitive to the intrinsic properties of substituents (Table 3). For instance, compound 3a displayed an extremely low *Φ* of 0.19% in solution, indicative of its almost non-emissive nature, while the introduction of either an aldehyde group^46^ (3c) or a sulfur-containing^47^ moiety (3d) completely quenched the emission, leading to quantum yields approaching zero. In contrast, compounds 4a and 4c exhibited remarkably high quantum yields of 69.80% and 67.37% in solution states, respectively, while the presence of a sulfur-containing heterocycle in 4b resulted in a lower quantum yield of 20.79%. In the solid state, the quantum yields were found to decrease compared to their solution-state counterparts. Compound 4a exhibited a quantum yield of 49.30%, while those of 4b and 4c decreased to 0% and 16.66%, respectively, upon the incorporation of sulfur or fluorine atoms. Collectively, these results underscore the pivotal role of BN unit positioning and substituent effects in modulating the photophysical behaviour of these systems, offering valuable guidance for the rational design of high-performance organic emitters.

Organic Room-Temperature Phosphorescence (ORTP) materials^48^ have attracted growing interest attributable to their potential applications in bioimaging,^49–52^ sensing,^53,54^ and optical displays.^55–57^ Their favorable features—including low cytotoxicity, structural tunability, long-lived emission, high signal-to-noise ratios, minimal background fluorescence, and strong resistance to light scattering—make them particularly appealing for real-world applications. However, despite these advantages, ORTP materials often suffer from inherent drawbacks such as poor mechanical properties, limited processability, and high sensitivity to oxygen and moisture, which collectively hinder their integration into practical devices and large-scale applications. These challenges necessitate the development of strategies to improve their structural robustness, environmental stability, and fabrication compatibility. To address these limitations, incorporating ORTP luminophores into appropriate host matrices has proven to be an effective strategy for enhancing their mechanical strength, processability, and environmental stability. In particular, polymeric matrices can not only provide structural support but also create confined microenvironments that suppress nonradiative decay pathways and protect triplet excitons from quenching by oxygen and moisture. Accordingly, two representative BN-fused aromatic compounds, 3a and 4a, were selected as model systems to investigate their RTP behavior in different physical states and various polymer matrices, thereby validating the role of polymer hosts in modulating their emission properties.

**Fig. 4:**
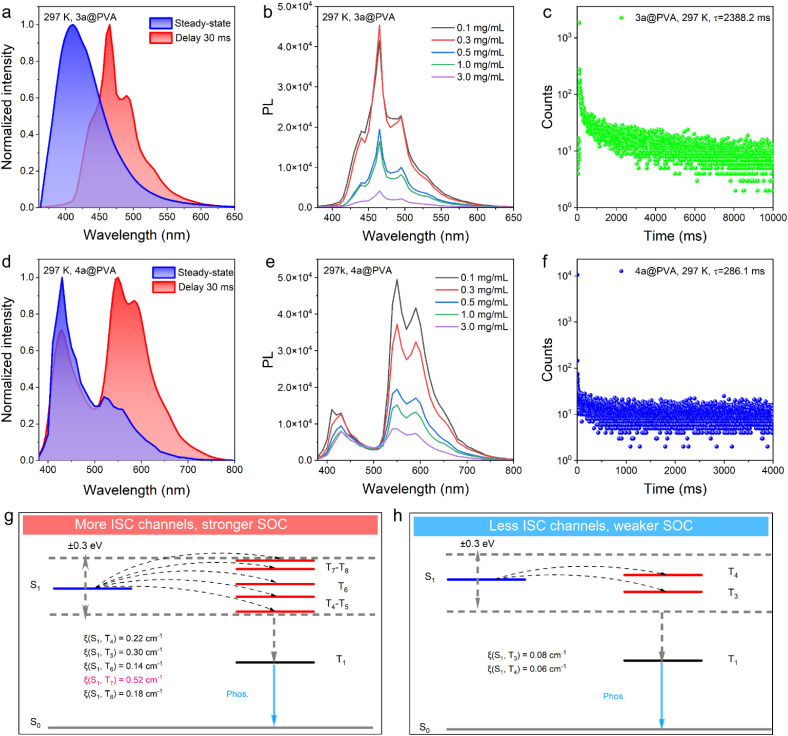
Photophysical properties of PVA-doped films 3a@PVA and 4a@PVA. (a and d) Prompt and delayed PL spectra. (b and e) Doping concentration-dependent delayed PL spectra of films 3a@PVA and 4a@PVA. (c and f) Time-resolved phosphorescence decay curves. (g) Energy-level diagrams and possible ISC channels from excited singlet states (S_1_) to excited triplet states (T_*n*_) for 3a (isolated). (h) Energy-level diagrams and possible ISC channels from excited singlet states (S_1_) to excited triplet states (T_*n*_) for 4a (isolated).

Systematic investigations revealed that, under ambient conditions, neither compound exhibits RTP in solution (Fig. S1 and S4). Moreover, compound 4a shows no RTP in both the solid state (Fig. S5) and crystalline states (Fig. S6), whereas 3a displays only a very weak phosphorescence signal in these states (Fig. S2 and S3). Furthermore, the phosphorescence lifetimes of both compounds could not be detected at room temperature in solution, solid states, or crystalline states, further confirming that RTP behaviours were not observable for 3a and 4a under these conditions. Previous studies have revealed that 3a adopts a non-planar twisted conformation^17^ (Fig. S16). Based on this structural feature, we hypothesized that incorporating 3a into a polymer matrix could effectively restrict molecular vibrations,^58^ thereby suppressing non-radiative decay pathways and enhancing RTP performance. Furthermore, both compounds contain polar B–N bonds that provide a robust structural basis for interaction with polymer matrices. The highly electronegative nitrogen atoms can form hydrogen bonds^59^ with hydroxyl groups, while the electron-deficient boron centers can coordinate with oxygen atoms *via* B–O bonds.^60,61^ Based on these considerations, polyvinyl alcohol (PVA), poly(methyl methacrylate) (PMMA), and polyvinylpyrrolidone (PVP) were selected as host matrices to evaluate the RTP properties of the two compounds at 297 K. The results demonstrate that both compounds exhibit RTP in all three matrices, albeit with significant differences in phosphorescence lifetimes. Specifically, compound 4a shows lifetimes of 286.1 ms, 408.8 ms, and 516.27 ms in PVA (Fig. S10), PMMA (Fig. S11), and PVP (Fig. S12), respectively, while compound 3a exhibits lifetimes of 2388.2 ms (Fig. S7), 8.82 ms (Fig. S8), and 669.96 ms (Fig. S9) in the corresponding matrices. Considering the overall RTP lifetimes and stability, PVA was chosen as the polymer matrix for further detailed investigations. The resulting films exhibited visually discernible blue (3a@PVA) and yellow (4a@PVA) afterglow emissions that persisted for several seconds under ambient conditions. Delayed emission spectra (Fig. 4a, e and Table 4) revealed marked red shifts relative to their prompt fluorescence, corroborating the dual-emissive nature of these systems—featuring both fluorescence and ultralong phosphorescence components. To further optimize and evaluate their performance, we systematically investigated the influence of doping concentration on RTP intensity. As shown in Fig. 4b and f, 3a@PVA exhibited maximum phosphorescence intensity at a doping level of 0.3 mg mL^−1^, whereas higher concentrations led to a gradual quenching of emission. A similar trend was observed for 4a@PVA. This concentration-dependent quenching is likely due to enhanced intermolecular interactions at elevated doping levels,^62^ which facilitate non-radiative deactivation pathways and suppress phosphorescence. In addition to concentration effects, temperature was found to play a critical role in modulating RTP behaviours. Time-resolved photoluminescence decay measurements were carried out across a range of temperatures, revealing that the phosphorescence lifetimes of both films decreased progressively with rising temperature (Fig. S13–S15). Notably, under ambient conditions, 3a@PVA exhibited an exceptional phosphorescence lifetime of 2388.2 ms (Fig. 4c), significantly longer than the 286.1 ms lifetime recorded for 4a@PVA (Fig. 4g). These findings highlight the profound influence of BN unit positioning on the RTP performance of the materials. The pronounced differences in their photoluminescent behaviors thereby prompted us to further elucidate the underlying mechanisms.

**Table 4:** Photophysical data of the doped PVA films at room temperature^a^

Entries	*λ*_f_ [nm]	*λ*_p_ [nm]	*Φ*_t_ [%]	*Φ*_f_ [%]	*Φ*_p_ [%]	〈*τ*〉_p_ [ms]	*k* ^p^_r_ [s^−1^]	*k* ^p^_nr_ [s^−1^]
3a@PVA	410	465	6.05	4.20	1.80	2388.2	0.0075	0.4110
4a@PVA	429	550	24.30	16.30	8.04	286.1	0.2800	3.2100

a *λ*_ex_ = 350 nm, delayed time = 30 ms. *Φ*_t_ = *Φ*_f_ + *Φ*_p_, *k*^p^_r_ = *Φ*_p_/〈*τ*〉_p_, and *k*^p^_nr_ = (1 − *Φ*_p_)/〈*τ*〉_p_. *λ*_f_ and *λ*_p_ are the emission maxima of fluorescence and phosphorescence of the crystals. *Φ*_t_, *Φ*_f_, and *Φ*_p_ are the quantum efficiencies of total emission, fluorescence, and phosphorescence of doped PVA, respectively.

Elucidating the light-emission process is essential for understanding the fundamental mechanisms underlying photoluminescence. The interplay between intersystem crossing (ISC) and spin–orbit coupling (SOC) plays a pivotal role in the generation of RTP. To qualitatively analyze the energy levels of singlet and triplet states, TD-DFT calculations were performed to map the singlet and triplet energy levels and evaluate potential ISC pathways. Efficient singlet-to-triplet (S_1_ → Tn) transition pathways were systematically identified by applying a stringent energy gap criterion, specifically requiring the singlet-triplet energy splitting (Δ*E*_ST_) to be within an absolute value of less than 0.3 eV.^63^ Specifically, compound 3a exhibits five effective ISC channels,^17^ facilitating transitions from the singlet excited state S_1_ to triplet states T_4_, T_5_, T_6_, T_7_ and T_8_ (Fig. 4g). In contrast, 4a features only two effective ISC pathways^17,39^ (Fig. 4h). Further analysis of SOC values provided mechanistic insights into the distinct photophysical behaviours of the two compounds: 3a displays SOC values ranging from 0.18 cm^−1^ to a maximum of 0.52 cm^−1^ (Fig. 4g and Table S1), which are significantly higher than those of 4a, whose maximum SOC value reaches only 0.08 cm^−1^ (Fig. 4h and Table S2). These results indicate that, both in terms of the number of effective ISC channels and the SOC values, compound 3a is more favourable for the generation of triplet excitons, which serves as a prerequisite for achieving RTP.^64^

**Fig. 5:**
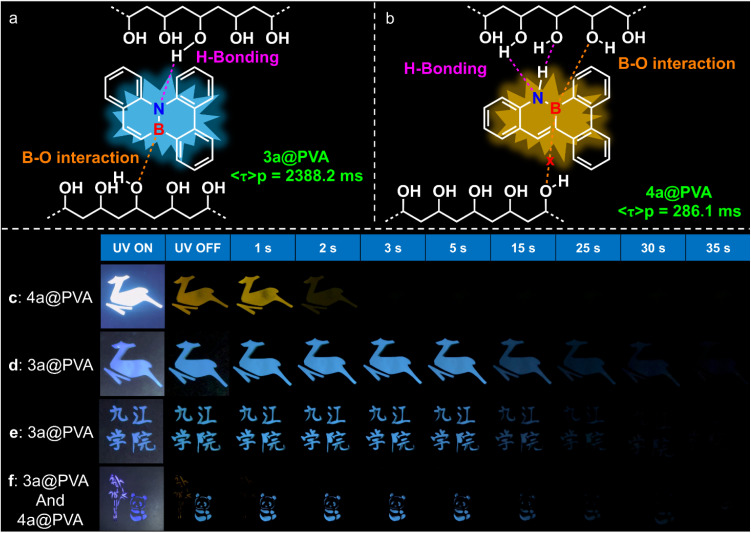
(a and b) Possible schematic models of the interactions of 3a and 4a with PVA, respectively. (c–f) Potential applications of doping the PVA system in the fields of anti-counterfeiting and information encryption. (c) was excited under UV irradiation at a wavelength of 365 nm with a power of 10 W, while (d–f) were excited using a 10 W UV lamp at 265 nm.

It is of significant importance to elucidate the key factors responsible for the marked differences in RTP behaviour exhibited by 3a and 4a, despite their structural isomerism. To rationalise this phenomenon, a schematic illustration of the possible interactions between the compounds and the PVA matrix is proposed. It is evident that both 3a and 4a contain nitrogen (N) and boron (B) atoms, which facilitates the formation of hydrogen bonds^59^ and B–O coordination interactions^60,61^ with the PVA matrix. This effectively stabilises the system. However, a marked discrepancy in the spatial distribution of these interactions is evident between the two systems. In the 3a@PVA system, the N and B atoms are positioned on opposite sides of the molecule, enabling bilateral anchoring within the PVA matrix (Fig. 5a). This spatial constraint significantly restricts intramolecular motions, effectively suppressing nonradiative decay pathways and thereby enhancing the RTP performance. In contrast, the 4a@PVA system exhibits an asymmetric anchoring configuration, with both the N and B atoms located on the same side of the molecule (Fig. 5b). In this case, the N and B atoms preferentially interact with hydroxyl groups in the less sterically hindered regions of the PVA matrix through hydrogen bonding and B–O coordination, reaching a saturated coordination state. As a result, further interactions with PVA chains on the more sterically congested side are effectively impeded. Consequently, the molecular motions of 4a are less constrained, resulting in enhanced nonradiative decay processes and significantly reduced emission efficiency. Moreover, the lower rigidity associated with this asymmetric structure facilitates vibrational relaxation, thereby promoting non-radiative energy dissipation. Experimental data further support our hypothesis. As shown in Table 4, the phosphorescence radiative decay rate (*k*^p^_r_) and nonradiative decay rate (*k*^p^_nr_) of 4a@PVA are 37 times and 7.8 times higher, respectively, than those of 3a@PVA. These findings indicate that the deactivation of triplet excitons in 3a@PVA is significantly suppressed, directly contributing to its prolonged RTP lifetime.

To further assess the practical applicability of the synthesized materials in visual information display, we conducted patterning and phosphorescence decay imaging experiments using PVA-doped films of 3a and 4a (denoted as 3a@PVA and 4a@PVA, respectively). The 3a@PVA film successfully reproduced a well-defined “deer” pattern (Fig. 5d and Video S2) as well as the Chinese characters for “Jiujiang University” (Fig. 5e and Video S3), both of which exhibited a vivid blue afterglow visible to the naked eye for up to 30 seconds. In contrast, the 4a@PVA film enabled the generation of a similar “deer” pattern (Fig. 5c and Video S1), emitting a yellow afterglow with a markedly shorter lifetime of approximately 2 seconds. These results are in excellent agreement with the phosphorescence lifetimes measured previously. To explore potential applications in optical anti-counterfeiting, we designed a composite pattern featuring “a panda under bamboo,” in which the bamboo was rendered using 4a@PVA and the panda using 3a@PVA (Fig. 5f and Video S4). The pronounced differences in emission color and afterglow duration between the two films enabled the pattern to be temporally resolved upon cessation of excitation, thus demonstrating a robust time-gated luminescence encoding strategy. Taken together, these findings highlight the considerable promise of 3a@PVA and 4a@PVA as advanced phosphorescent materials for applications in visual display technologies, dynamic patterning, and information security.

## Conclusions

In this study, we developed a regioselective synthetic strategy for the construction of 2,1-BN naphthalene derivatives using various *ortho*-halogenated phenylboronic acids, enabling the controlled synthesis of representative BN-fused isomers. The broad substrate compatibility underscores the generality and efficiency of this approach. Photophysical investigations revealed that both isomers exhibit RTP when embedded in a polyvinyl alcohol (PVA) matrix. Notably, the 3a@PVA film demonstrated outstanding RTP performance, featuring an ultralong phosphorescence lifetime of 2388.2 ms and a visible afterglow lasting up to 30 seconds. This pronounced difference can be attributed to the presence of more efficient ISC channels, larger SOC values, and the more stable interactions between the 3a molecule and the PVA matrix, which collectively endow the material with enhanced potential for anti-counterfeiting applications. Overall, this work not only offers an efficient and versatile synthetic route to structurally defined BN isomers but also establishes a robust, patternable RTP platform, laying a foundation for the functional development of BN-based phosphorescent materials.

## Author contributions

Qiang Feng and Jianhua Liu conceptualized the project methodology, supervised the investigation, collected experimental data, built the data pipeline, analysed the data and wrote the original draft. Junxiong Yao collected experimental data, built the data pipeline and analysed the data. Yang Qiu, Zicheng Wang, Xia Wang, Weilin Chen, and Qianxin Wu collected experimental data and analysed the data. Xiaohua Cao, Jianqi Sun and Qianqian Ye built the data pipeline. Huanan Huang, Jianguo Wang and Dianyuan Wang conceptualized the project methodology, supervised the investigation and co-wrote the manuscript.

## Conflicts of interest

The authors declare no competing financial interest.

## Supplementary Material

SC-016-D5SC05061H-s001

SC-016-D5SC05061H-s002

SC-016-D5SC05061H-s003

SC-016-D5SC05061H-s004

SC-016-D5SC05061H-s005

## Data Availability

All data supporting the findings of this study are provided in the main text and the SI. The supplementary information (SI) for this article contains all relevant experimental details, including the synthesis procedures of the compounds, preparation methods of doping films, characterization data for the products, photoluminescence (PL) studies, density functional theory (DFT) calculations, and NMR spectroscopic data. See DOI: https://doi.org/10.1039/d5sc05061h.
