# Supplementary material for: Regioselective construction of two isomeric BN-fused aromatic frameworks enabling the synthesis of ultralong room-temperature phosphorescence materials
Source: Chem Sci. 2025 Aug 25;16(37):17470–9. doi: 10.1039/d5sc05061h (PMC12406039; doi:10.1039/d5sc05061h)
Supplement: SC-016-D5SC05061H-s001 [file SC-016-D5SC05061H-s001.pdf]

Supplementary Information (SI) for Chemical Science.
This journal is © The Royal Society of Chemistry 2025

Supporting Information

Regioselective Construction of Two Isomeric BN-Fused Aromatics Frameworks Enabling Ultralong Room-Temperature Phosphorescence Materials

Qiang Feng[a †], Junxiong Yao[a b †], Qianxin Wu[a], Yang Qiu[a], Zicheng Wang[a], Xia Wang[a], Weilin Chen[a], Sibo Tong[a], Xiaohua Cao[a], Jianqi Sun[a], Qianqian Ye, Jianhua Liu*[a], Dianyuan Wang*[a], Jianguo Wang*[b], Huanan Huang*[a]

[a] College of Chemistry and Chemical Engineering; Jiangxi Province Engineering Research Center of Ecological Chemical Industry, Jiujiang University, Jiujiang 332005, China. E-mail: huanan200890@163.com.

[b] College of Chemistry and Chemical Engineering, College of Green Chemistry and Environment, Institutes of Biomedical Sciences, Inner Mongolia Key Laboratory of Synthesis and Application of Organic Functional Molecules, Inner Mongolia University, Hohhot 010021, P. R. China, E-mail: wangjg@iccas.ac.cn

[†] These authors contributed equally.

Table of Contents

1. General information 2

2. General procedures...2

2.1 General Procedure for the Synthesis of 3...2

2.2 General Procedure for the Synthesis of 4...3

3. Preparation methods of doping films 3

4. Characterization data for products 4

5. PL Studies of Compound 3a-3f and 4a-4c 8

6. DFT Calculatuins 9

7. Reference...10

8. NMR spectroscopic data 10

1. General information

All chemicals were purchased from Adamas Reagent, Ltd, Energy chemical company, J&K Scientific Ltd, Alfa Aesar chemical company and so forth. Unless otherwise stated, all experiments were conducted in a seal tube under air atmosphere. Reactions were monitored by TLC or GC-MS analysis. Flash column chromatography was performed over silica gel (200-300 mesh).

^{1}H-NMR and ^{13}C-NMR spectra were recorded in $CDCl_3$ on a Bruker Avance 400 spectrometer (400 MHz ^{1}H, 101 MHz ^{13}C) at room temperature. Chemical shifts were reported in ppm on the scale relative to $CDCl_3$ (δ = 7.26 for ^{1}H-NMR, δ = 77.00 for ^{13}C-NMR) as an internal reference. High resolution mass spectra were recorded using Q-TOF time-of-flight mass spectrometer. Coupling constants (J) were reported in Hertz (Hz). The starting materials indoles were purchased from Bide Pharmatech Ltd. The reactant boronic acids were purchased from Bide Pharmatech Ltd and Energy chemical company. 2-(2-bromophenyl)-1,2-dihydrobenzo[e][1,2]azaborinine (**1**) were prepared according to the known methods reported by previous literatures[1, 2].

UV-vis absorption spectra were recorded on a SHIMADZU UV-2600i spectrophotometer. Photoluminescence spectra were recorded on a HITACHI F-4700 fluorescence spectrophotometer. The absolute fluorescence quantum yield was measured using a Hamamatsu quantum yield spectrometer Quantaurus QY (model C11347-11).

The geometries of 3a and 4a were energetically optimized by density functional theory (DFT) using the B3LYP density functional and the Def2SVP basis set[3, 4]. Time-dependent DFT (TD-DFT) was utilized at the same level of theory to calculate optimized singlet and triplet geometries. The above-mentioned quantum chemical calculations were carried out by using Gaussian 09. The spin-orbit coupling (SOC) constants were studied with PySOC package under the same functional/basis set based on their ground state geometry.

2. General procedures

2.1 General Procedure for the Synthesis of 3.

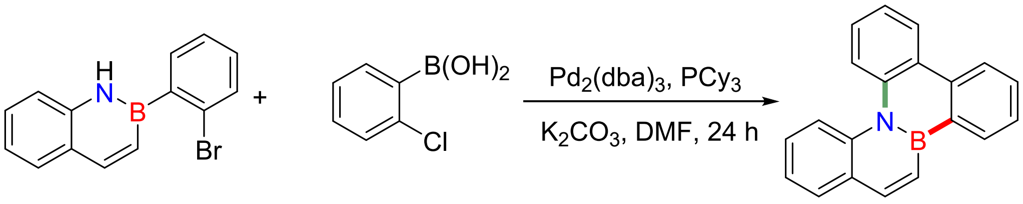

1a **2a** **3a**

To an oven-dried schlenk tube with a stir bar was added **1a** (0.2 mmol), (2-chlorophenyl)boronic acid (1 equiv), $Pd(dba)_2$ (10 mol %), PCy_3 (20 mol%), and K_2CO_3 (3 equiv). The tube was sealed with schlenk system, evacuated under vacuum, and purged with Ar three times. DMF (1 mL) was added, The resulting mixture was heated to 160°C and stirred 24 h. The reaction mixture was cooled to room temperature, and the resulting mixture was washed with water before being extracted with ethyl acetate. The combined organic phases were then dried over anhydrous sodium sulfate. The combined organic phases were concentrated under vacuum to obtain the crude product, which was then purified by flash column chromatography on silica gel using hexanes and dichloromethane as eluents.

2.2 General Procedure for the Synthesis of 4.

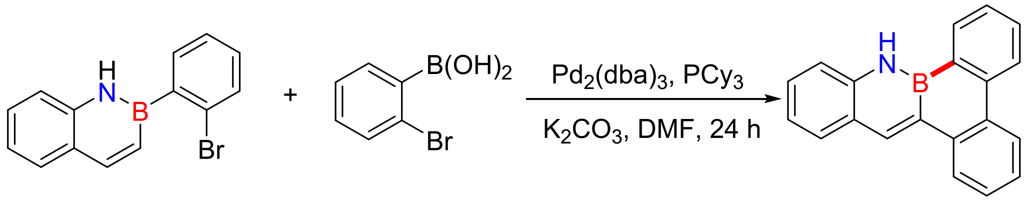

1a **2b** **4a**

To an oven-dried schlenk tube with a stir bar was added **1a** (0.2 mmol), (2-bromophenyl)boronic acid (1 equiv), $Pd(dba)_2$ (10 mol %), PCy_3 (20 mol%), and K_2CO_3 (3 equiv). The tube was sealed with schlenk system, evacuated under vacuum, and purged with Ar three times. DMF (1 mL) was added, The resulting mixture was heated to 160°C and stirred 24 h. The reaction mixture was cooled to room temperature, and the resulting mixture was washed with water before being extracted with ethyl acetate. The combined organic phases were then dried over anhydrous sodium sulfate. The combined organic phases were concentrated under vacuum to obtain the crude product, which was then purified by flash column chromatography on silica gel using hexanes and dichloromethane as eluents.

3. Preparation methods of doping films

To prepare doped polyvinyl alcohol (PVA) films with a doping concentration of 0.3 mg/mL as an example, 3.0 g of PVA powder was dissolved in 100 mL of deionized water with stirring at 100 °C until complete dissolution was achieved. An organic compound (1.5 mg) was dissolved in 1 mL of ethanol to form a clear solution. This solution was then added to 5 mL of the PVA solution and stirred to obtain a homogeneous clear solution. The resulting mixture was drop-cast onto a preheated quartz substrate and subjected to post-thermal annealing at 65 °C for 3 hours.

4. Characterization data for products

dibenzo[c,e]benzo[5,6][1,2]azaborinino[1,2-a][1,2]azaborinine (3a)

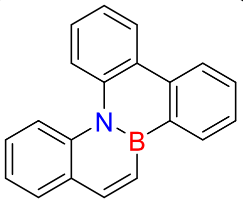

3a

The reaction was performed following the general procedure. The residue was purified by flash column chromatograph (silica gel, petroleum ether: AcOEt = 15:1, v/v) to give the product as a white solid (47 mg, 85%).^{1}H NMR (400 MHz, $CDCl_3$) δ 8.51 – 8.32 (m, 5H), 8.14 (d, J = 11.4 Hz, 1H), 7.81 – 7.72 (m, 2H), 7.69 – 7.55 (m, 2H), 7.47 – 7.37 (m, 3H), 7.34 – 7.27 (m, 1H).^{13}C NMR (101 MHz, $CDCl_3$) δ 144.5, 139.0, 137.6, 137.5, 133.5, 131.1, 129.8, 129.0, 128.2, 126.8, 126.7, 126.1, 125.7, 123.7, 122.4, 122.3, 122.1, 120.6. HRMS (ESI, m/z) calcd for $C_{20}H_{15}BN$ $[M+H]^+$: 280.1292; found: 280.1297.

14-methyldibenzo[c,e]benzo[5,6][1,2]azaborinino[1,2-a][1,2]azaborinine (3b)

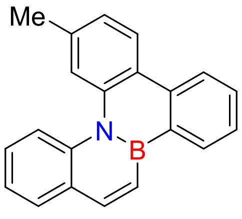

3b

The reaction was performed following the general procedure. The residue was purified by flash column chromatograph (silica gel, petroleum ether: AcOEt = 15:1, v/v) to give the product as a white solid (44 mg, 75%). ^{1}H NMR (400 MHz, $CDCl_3$) δ 8.49 – 8.44 (m, 1H), 8.40 (d, J = 8.5 Hz, 1H), 8.36 (d, J = 8.1 Hz, 1H), 8.29 (d, J = 8.2 Hz, 1H), 8.21 – 8.09 (m, 2H), 7.81 – 7.72 (m, 2H), 7.65 (d, J = 11.4 Hz, 1H), 7.60 – 7.55 (m, 1H), 7.47 – 7.41 (m, 1H), 7.34 – 7.28 (m, 1H), 7.25 – 7.18 (m, 1H), 2.48 (s, 3H). ^{13}C NMR (101 MHz, $CDCl_3$) δ 144.4, 139.1, 137.7, 137.4, 136.0, 133.5, 131.1, 129.8, 129.1, 126.7, 126.3, 125.8, 125.6, 124.8, 122.5, 122.3, 121.9, 120.7, 21.6. HRMS (ESI, m/z) calcd for $C_{21}H_{17}BN$ $[M+H]^+$: 294.1449; found: 294.1453.

dibenzo[c,e]benzo[5,6][1,2]azaborinino[1,2-a][1,2]azaborinine-15-carbaldehyde (3c)

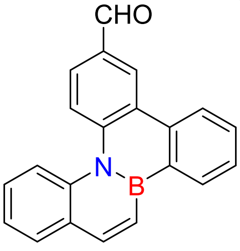

3c

The reaction was performed following the general procedure. The residue was purified by flash column chromatograph (silica gel, petroleum ether: AcOEt = 15:1, v/v) to give the product as a white solid (14 mg, 23%). ^{1}H NMR (400 MHz, $CDCl_3$) δ 10.15 (d, J = 5.7 Hz, 1H), 8.89 (d, J = 5.6 Hz, 1H), 8.44 (q, J = 7.7 Hz, 3H), 8.29 – 8.07 (m, 2H), 7.93 – 7.72 (m, 3H), 7.72 – 7.53 (m, 2H), 7.50 – 7.30 (m, 2H). ^{13}C NMR (101 MHz, $CDCl_3$) δ 191.3, 145.2, 142.0, 138.4, 136.8, 133.7, 131.7, 131.6, 130.0, 129.2, 128.7, 128.3, 127.4, 127.1, 126.2, 123.2, 122.8, 122.2, 120.7. HRMS (ESI, m/z) calcd for $C_{21}H_{14}BNNaO$ $[M+Na]^+$: 330.1061; found: 330.1071.

benzo[c]benzo[5,6][1,2]azaborinino[1,2-a]thieno[3,2-e][1,2]azaborinine (3d)

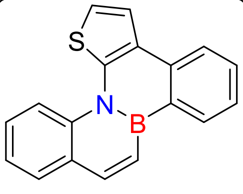

3d

The reaction was performed following the general procedure. The residue was purified by flash column chromatograph (silica gel, petroleum ether: AcOEt = 15:1, v/v) to give the product as a yellow solid (31 mg, 55%). ^{1}H NMR (400 MHz, $CDCl_3$) δ 9.12 (d, J = 8.6 Hz, 1H), 8.63 (dd, J = 7.6, 1.4 Hz, 1H), 8.15 (dd, J = 9.7, 6.8 Hz, 2H), 7.89 – 7.74 (m, 4H), 7.69 – 7.61 (m, 1H), 7.60 – 7.53 (m, 1H), 7.47 – 7.41 (m, 1H), 7.17 (d, J = 5.8 Hz, 1H). ^{13}C NMR (101 MHz, $CDCl_3$) δ 143.4, 141.3, 138.9, 136.7, 134.1, 131.2, 130.5, 128.5, 128.4, 127.7, 125.3, 123.3, 122.6, 122.5, 117.6, 116.1. HRMS (ESI, m/z) calcd for $C_{21}H_{15}BNO$ $[M+H]^+$: 286.0856; found: 286.0865.

2-fluorodibenzo[c,e]benzo[5,6][1,2]azaborinino[1,2-a][1,2]azaborinine (3e)

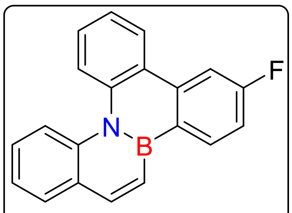

3e

The reaction was performed following the general procedure. The residue was purified by flash column chromatograph (silica gel, petroleum ether: AcOEt = 15:1, v/v) to give the product as a white solid (30 mg, 50%). ^{1}H NMR (400 MHz, $CDCl_3$) δ 8.36 (dd, J = 8.3, 6.5 Hz, 1H), 8.32 – 8.18 (m, 3H), 8.05 (d, J = 11.4 Hz, 1H), 7.95 (dd, J = 11.3, 2.4 Hz, 1H), 7.75 – 7.66 (m, 1H), 7.50 (d, J = 11.4 Hz, 1H), 7.41 – 7.29 (m, 3H), 7.28 – 7.18 (m, 2H). ^{13}C NMR (101 MHz, $CDCl_3$) δ 166.5, 164.0, 144.6, 140.1, 138.8, 137.8, 136.0, 129.9, 129.0, 127.2, 126.8, 126.7, 125.7, 123.7, 122.5, 120.5, 114.4, 108.5. HRMS (ESI, m/z) calcd for $C_{20}H_{14}BFN$ $[M+H]^+$: 298.1198; found: 298.1197.

2,9-difluorodibenzo[c,e]benzo[5,6][1,2]azaborinino[1,2-a][1,2]azaborinine (3f)

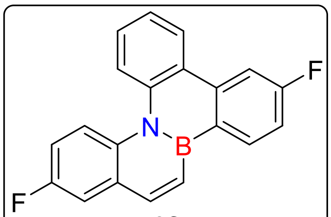

3f

The reaction was performed following the general procedure. The residue was purified by flash column chromatograph (silica gel, petroleum ether: AcOEt = 15:1, v/v) to give the product as a white solid (40 mg, 63%). ^{1}H NMR (400 MHz, $CDCl_3$) δ 8.37 (dd, J = 8.3, 6.5 Hz, 1H), 8.30 – 8.18 (m, 3H), 8.04 – 7.93 (m, 2H), 7.57 (d, J = 11.4 Hz, 1H), 7.43 – 7.33 (m, 3H), 7.29 – 7.21 (m, 1H), 7.15 – 7.06 (m, 1H). ^{13}C NMR (101 MHz, $CDCl_3$) δ 166.5, 164.0, 158.9, 156.5, 143.6, 140.0, 137.7, 135.9, 135.1, 130.1, 127.2, 127.2, 126.9, 125.8, 123.8, 122.1, 114.4, 108.5. HRMS (ESI, m/z) calcd for $C_{20}H_{13}BF_2N$ $[M+H]^+$: 316.1104; found: 316.1105.

10H-benzo[e]dibenzo[3,4:5,6]borinino[1,2-b][1,2]azaborinine (4a)

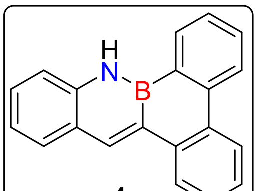

4a

The reaction was performed following the general procedure. The residue was purified by flash column chromatograph (silica gel, petroleum ether: AcOEt = 10:1, v/v) to give the product as a white solid (30 mg, 53%). ^{1}H NMR (400 MHz, $CDCl_3$) δ 8.97 (s, 1H), 8.67 (s, 1H), 8.49 (q, *J* = 7.6 Hz, 3H), 8.23 (d, *J* = 7.4 Hz, 1H), 7.91 (d, *J* = 7.9 Hz, 1H), 7.70 (t, *J* = 7.6 Hz, 1H), 7.60 – 7.46 (m, 5H), 7.32 (t, *J* = 7.3 Hz, 1H). ^{13}C NMR (101 MHz, $CDCl_3$) δ 141.3, 139.6, 136.9, 134.0, 133.4, 130.7, 130.4, 129.9, 128.7, 127.2, 127.2, 126.2, 125.6, 125.0, 124.2, 123.0, 121.2, 118.2. HRMS (ESI, m/z) calcd for $C_{20}H_{15}BN$ $[M+H]^+$: 280.1292; found: 280.1299.

9H-benzo[e]benzo[5,6]thieno[3',4':3,4]borinino[1,2-b][1,2]azaborinine (4b)

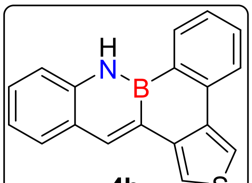

4b

The reaction was performed following the general procedure. The residue was purified by flash column chromatograph (silica gel, petroleum ether: AcOEt = 10:1, v/v) to give the product as a yellow solid (9 mg, 15%). ^{1}H NMR (400 MHz, $CDCl_3$) δ 8.75 (s, 1H), 8.41 – 8.27 (m, 2H), 8.12 – 8.01 (m, 2H), 7.94 (d, *J* = 2.6 Hz, 1H), 7.80 (d, *J* = 7.9 Hz, 1H), 7.47 (t, *J* = 7.3 Hz, 1H), 7.43 – 7.34 (m, 3H), 7.24 (d, *J* = 3.0 Hz, 1H). ^{13}C NMR (101 MHz, $CDCl_3$) δ 144.6, 139.4, 136.8, 133.3, 131.7, 130.3, 129.3, 128.5, 127.3, 126.8, 125.6, 125.6, 123.1, 121.2, 118.8, 117.9. HRMS (ESI, m/z) calcd for $C_{18}H_{13}BNS$ $[M+H]^+$: 286.0856; found: 286.0865.

13-fluoro-10H-benzo[e]dibenzo[3,4:5,6]borinino[1,2-b][1,2]azaborinine (4c)

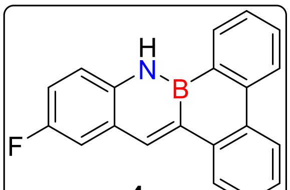

4c

The reaction was performed following the general procedure. The residue was purified by flash column chromatograph (silica gel, petroleum ether: AcOEt = 10:1, v/v) to give the product as a white solid (18 mg, 30%). ^{1}H NMR (400 MHz, $CDCl_3$) δ 8.75 (s, 1H), 8.41 – 8.27 (m, 2H), 8.12 – 8.01 (m, 2H), 7.94 (d, *J* = 2.6 Hz, 1H), 7.80 (d, *J* = 7.9 Hz, 1H), 7.47 (t, *J* = 7.3 Hz, 1H), 7.43 – 7.34 (m, 3H), 7.24 (d, *J* = 3.0 Hz, 1H). ^{13}C NMR (101 MHz, $CDCl_3$) δ 144.6, 139.4, 136.8, 133.3, 131.7, 130.3, 129.3, 128.5, 127.3, 126.8, 125.6, 125.6, 123.1, 121.2, 118.8, 117.9. HRMS (ESI, m/z) calcd for $C_{20}H_{14}BFN$ $[M+H]^+$: 298.1198; found: 298.1197.

5. PL Studies of Compound 3a and 4a

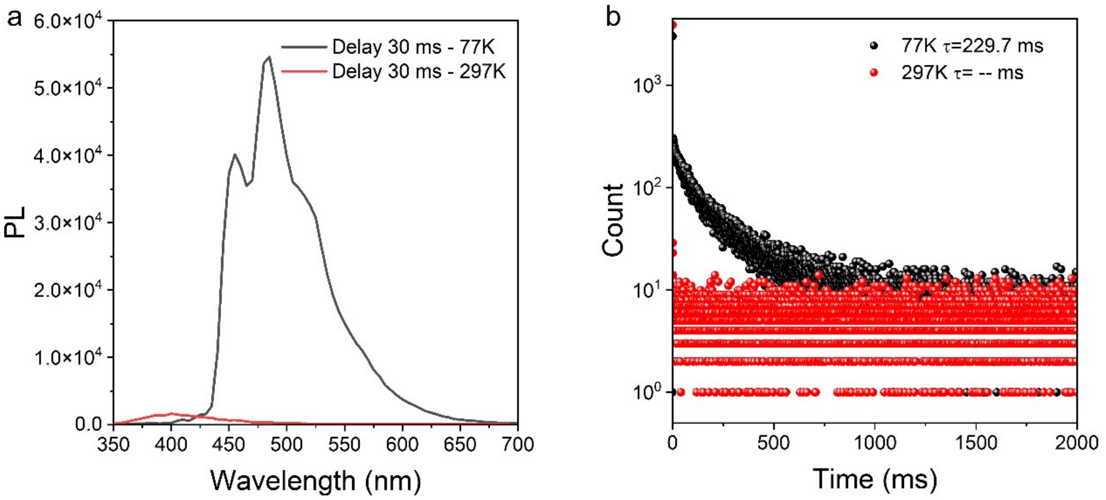

Figure S1. (a) Temperature-dependent phosphorescence spectra of **3a** in ethanol solution. (b) Time-resolved phosphorescence decay curves of **3a** in ethanol solution.

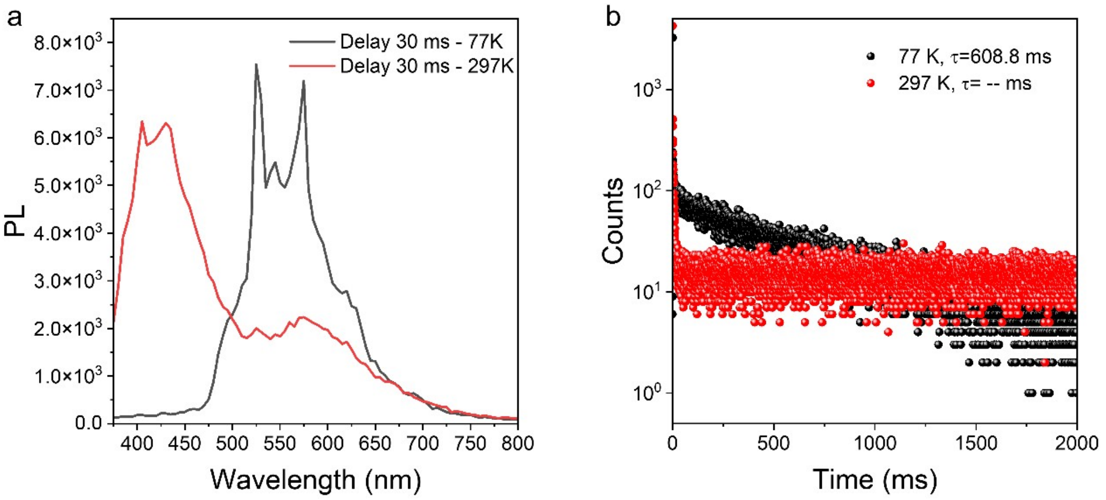

Figure S2. (a) Temperature-dependent phosphorescence spectra of **3a** in solid state. (b) Time-resolved phosphorescence decay curves of **3a** in solid state.

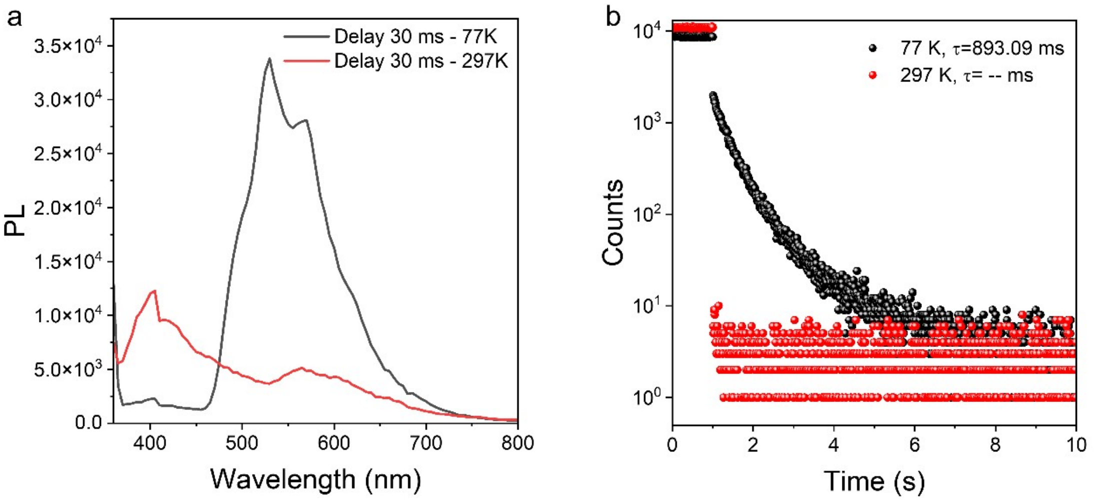

Figure S3. (a) Temperature-dependent phosphorescence spectra of **3a** in crystalline state. (b) Time-resolved phosphorescence decay curves of **3a** in crystalline state.

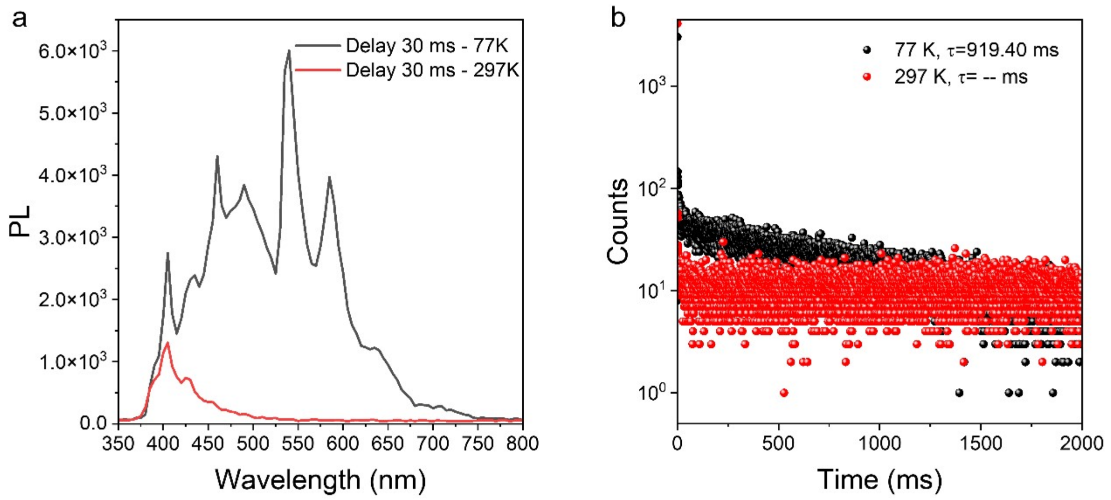

Figure S4. (a) Temperature-dependent phosphorescence spectra of **4a** in ethanol solution. (b) Time-resolved phosphorescence decay curves of **4a** in ethanol solution.

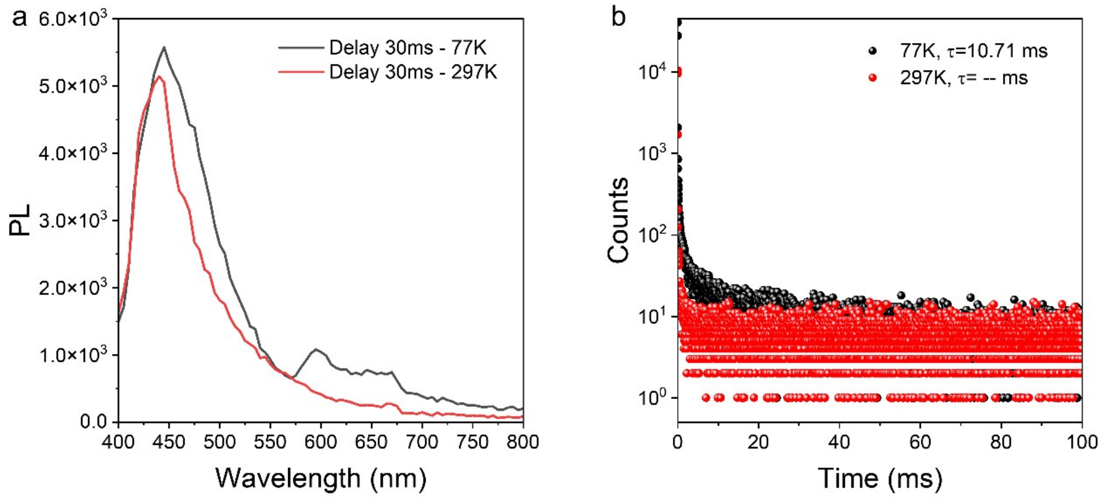

Figure S5. (a) Temperature-dependent phosphorescence spectra of **4a** in solid state. (b) Time-resolved phosphorescence decay curves of **4a** in solid state.

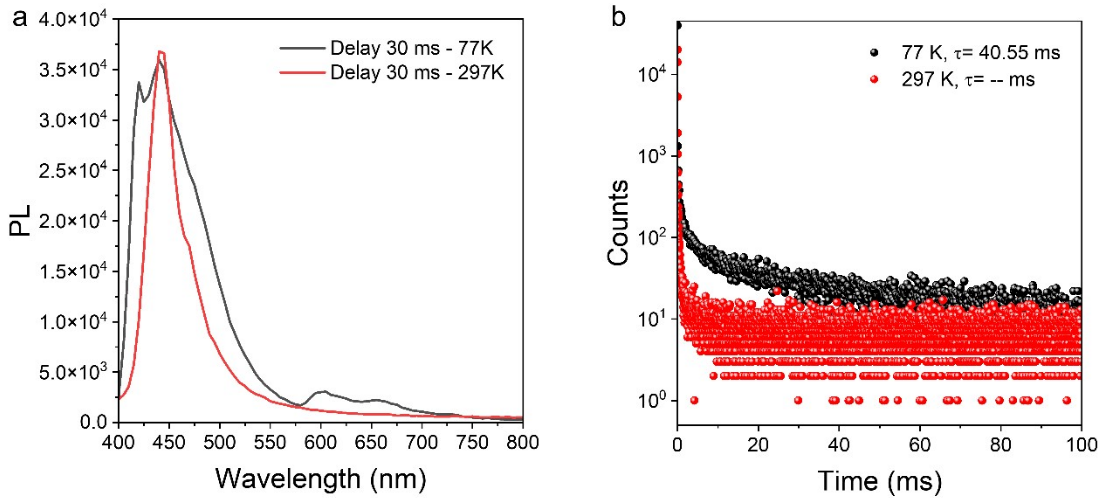

Figure S6. (a) Temperature-dependent phosphorescence spectra of **4a** in crystalline state. (b) Time-resolved phosphorescence decay curves of **4a** in crystalline state.

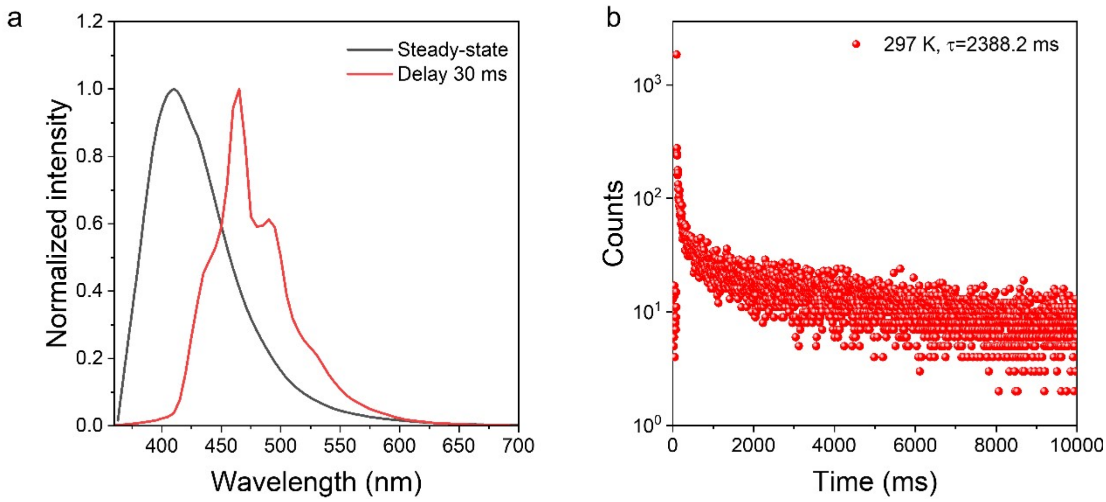

Figure S7. Photophysical properties of doping PVA film **3a@PVA**. (a) Prompt and delayed PL spectra. (b) Time-resolved phosphorescent decay curves.

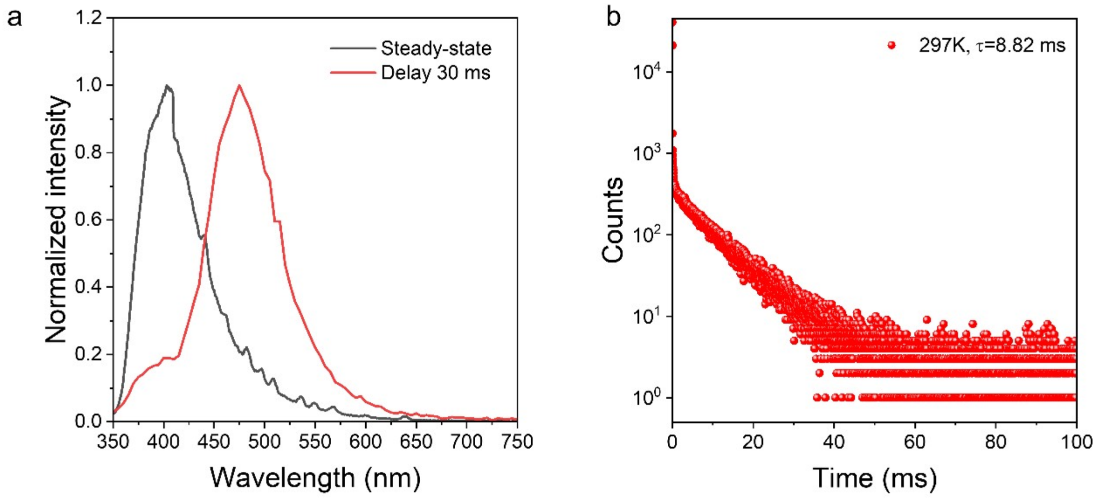

Figure S8. Photophysical properties of doping PMMA film **3a@PMMA**. (a) Prompt and delayed PL spectra. (b) Time-resolved phosphorescent decay curves.

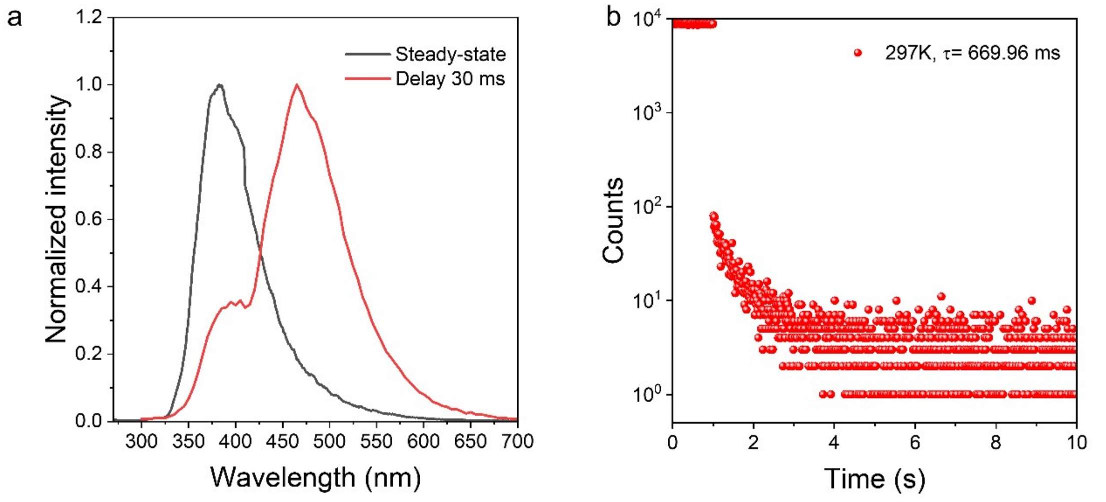

Figure S9. Photophysical properties of doping PVP film **3a@PVP**. (a) Prompt and delayed PL spectra. (b) Time-resolved phosphorescent decay curves.

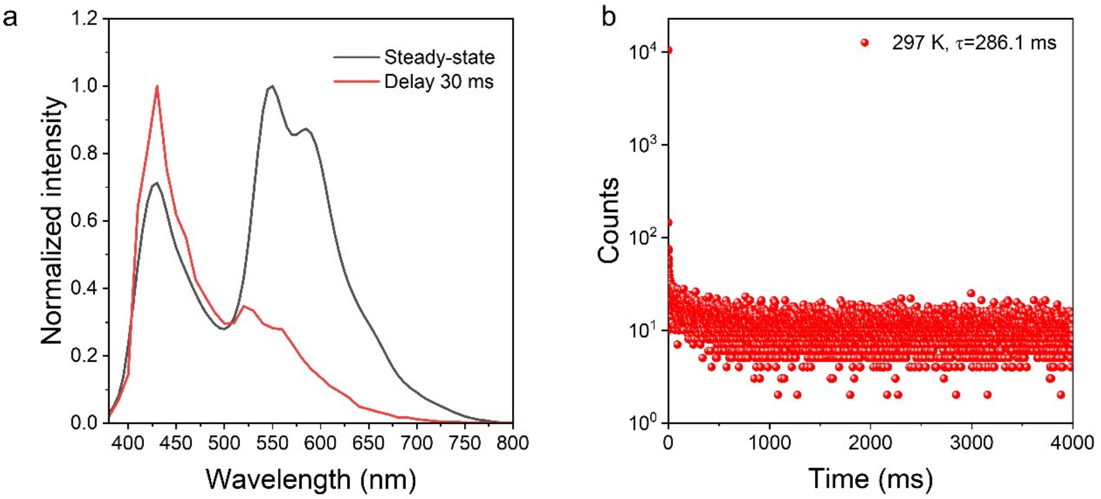

Figure S10. Photophysical properties of doping PVA film **4a@PVA**. (a) Prompt and delayed PL spectra. (b) Time-resolved phosphorescent decay curves.

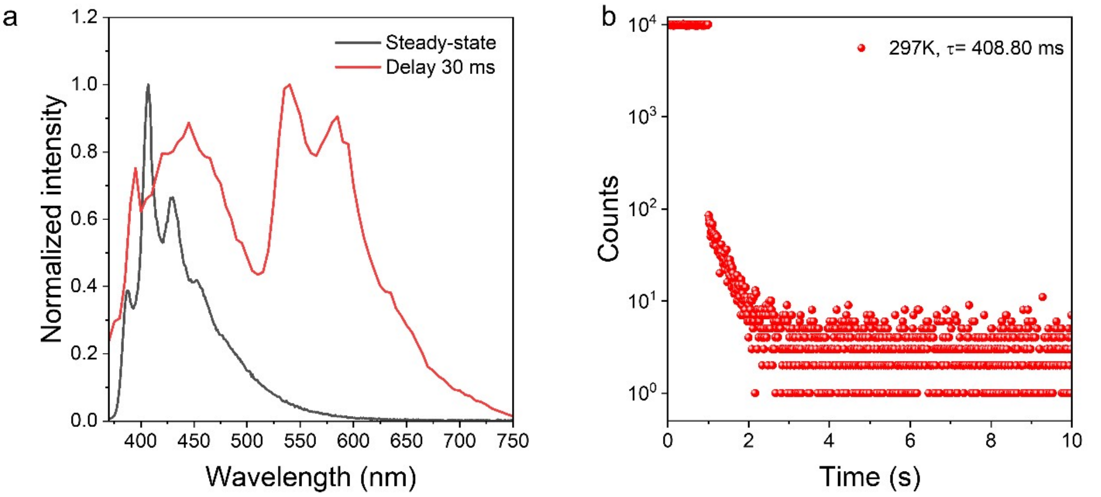

Figure S11. Photophysical properties of doping PMMA film **4a@PMMA**. (a) Prompt and delayed PL spectra. (b) Time-resolved phosphorescent decay curves.

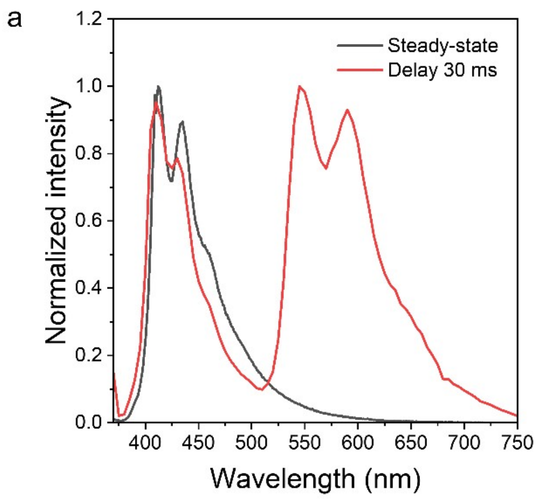

Figure S12. Photophysical properties of doping PVP film **4a@PVP**. (a) Prompt and delayed PL spectra. (b) Time-resolved phosphorescent decay curves.

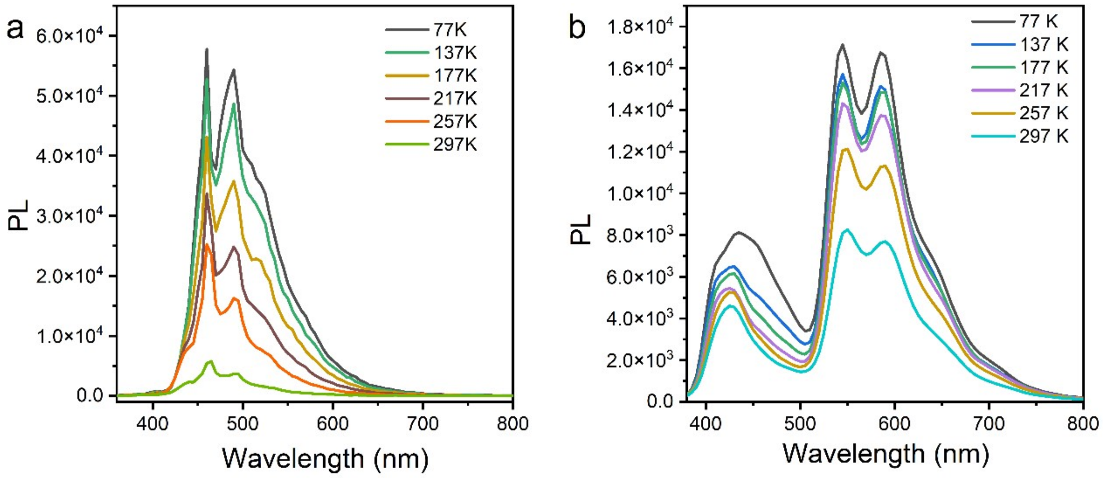

Figure S13. Temperature-dependent phosphorescence spectra of **3a@PVA** (a) and **4a@PVA** (b).

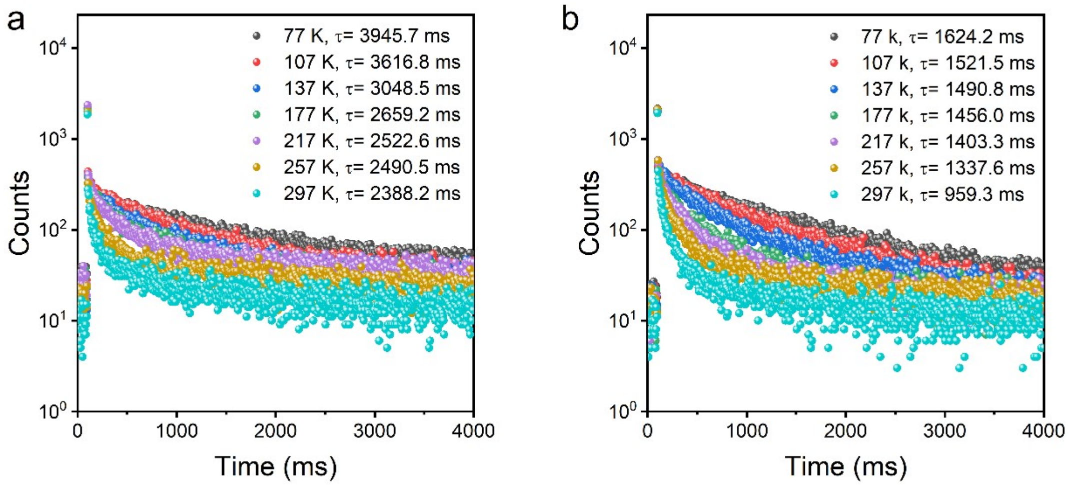

Figure S14. Time-resolved phosphorescence decay curves of **3a@PVA** at various temperatures, with emission wavelengths of 460 nm (a) and 490 nm (b), respectively.

Figure S15. Time-resolved phosphorescence decay curves of 4a@PVA at various temperatures, with emission wavelengths of 550 nm (a) and 600 nm (b), respectively.

6. Packing patterns and intermolecular interactions

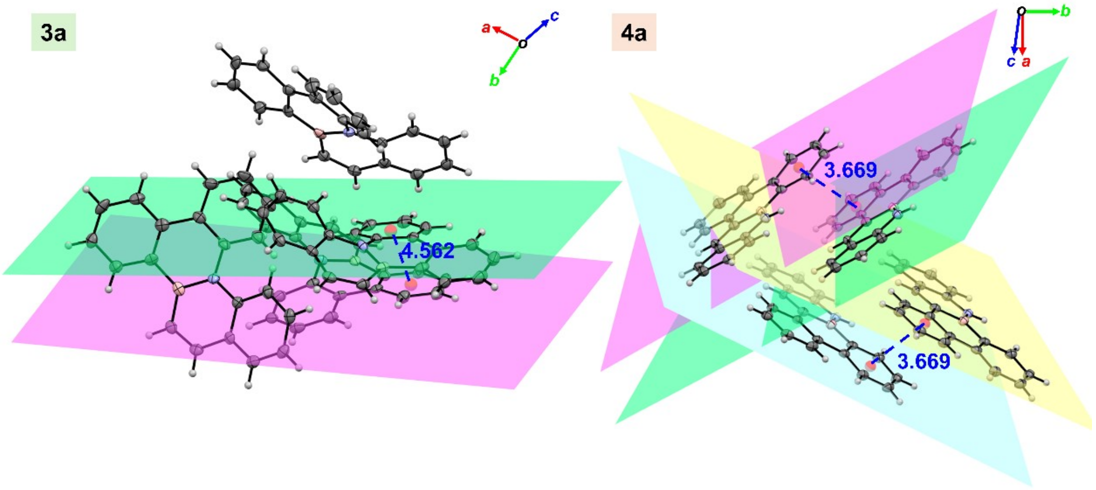

Figure S16. The packing patterns of **3a** and **4a.**

7. DFT Calculatuins

Table S1. The singlet and triplet excited state transition configurations of **3a** revealed by TD-DFT calculations. The matched excited states that $|S_1\text{-}T_n| < 0.3$ eV were highlighted in red.

3a (monomeric)	n-th	Energy（eV）	SOC（ξ, in cm^{-1}）
S_n	1	3.7836	
T_n	1	3.0016	0.99
	2	3.0643	0.22
	3	3.2401	0.22

	4	3.5390	0.22
	5	3.6972	0.30
	6	3.8319	0.14
	7	3.9900	0.52
	8	4.0799	0.18
	9	4.2343	0.44

Table S2. The singlet and triplet excited state transition configurations of **4a** revealed by TD-DFT calculations. The matched excited states that $|S_1\text{-}T_n| < 0.3$ eV were highlighted in red.

4a (monomeric)	n-th	Energy（eV）	SOC（ξ, in cm^{-1}）
S_n	1	3.4165	
T_n	1	2.3282	0.00
	2	2.9186	0.19
	3	3.2517	0.08
	4	3.4773	0.06
	5	3.7309	0.04
	6	3.7929	0.08
	7	3.8927	0.04

8. Reference

1. S. R. Wisniewski, C. L. Guenther, O. A. Argintaru and G. A. Molander, *J. Org. Chem.*, 2014, **79**, 365-378.
2. H. N. Huang, Y. Zhou, Y. W. Wang, X. H. Cao, C. Han, G. C. Liu, Z. X. Xu, C. C. Zhan, H. N. Hu, Y. Peng, P. Yan and D. P. Cao, *J. Mater. Chem. A*, 2020, **8**, 22023-22031.
3. T. Lu and F. Chen, *J. Comput. Chem.*, 2012, **33**, 580-592.
4. F. Weigend and R. Ahlrichs, *Phys. Chem. Chem. Phys.*, 2005, **7**, 3297-3305.

9. NMR spectroscopic data

dibenzo[c,e]benzo[5,6][1,2]azaborinino[1,2-a][1,2]azaborinine (3a)

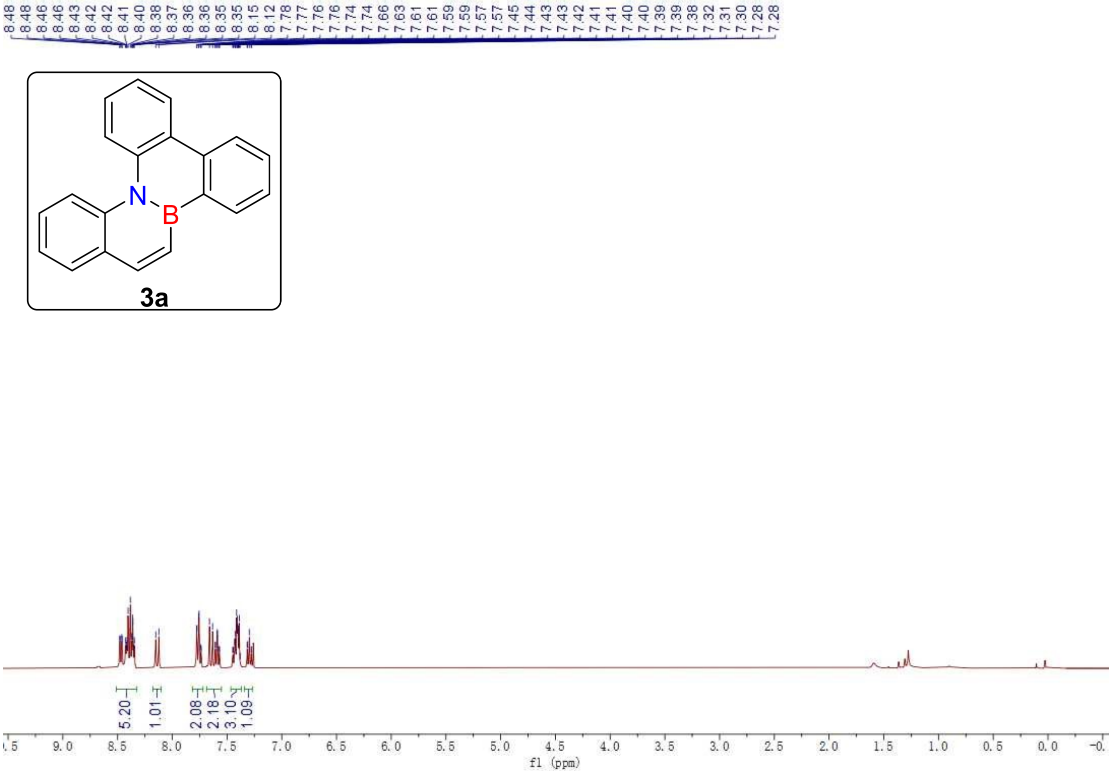

dibenzo[c,e]benzo[5,6][1,2]azaborinino[1,2-a][1,2]azaborinine (3a)

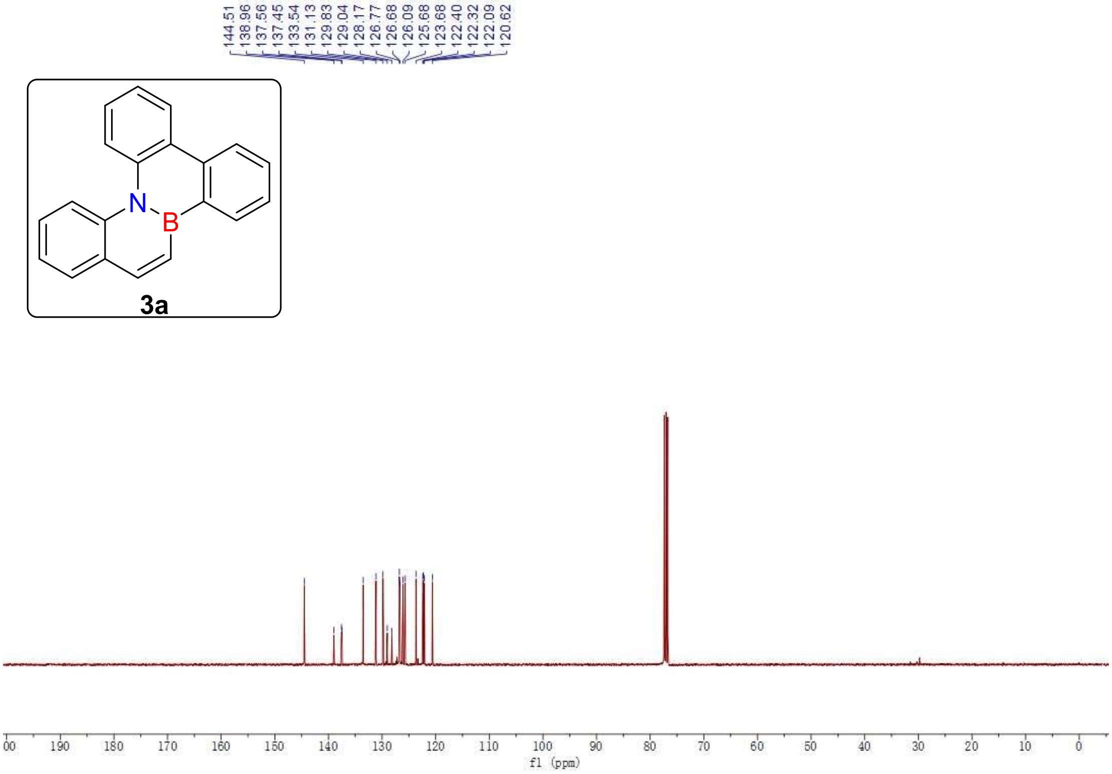

14-methyldibenzo[c,e]benzo[5,6][1,2]azaborinino[1,2-a][1,2]azaborinine (3b)

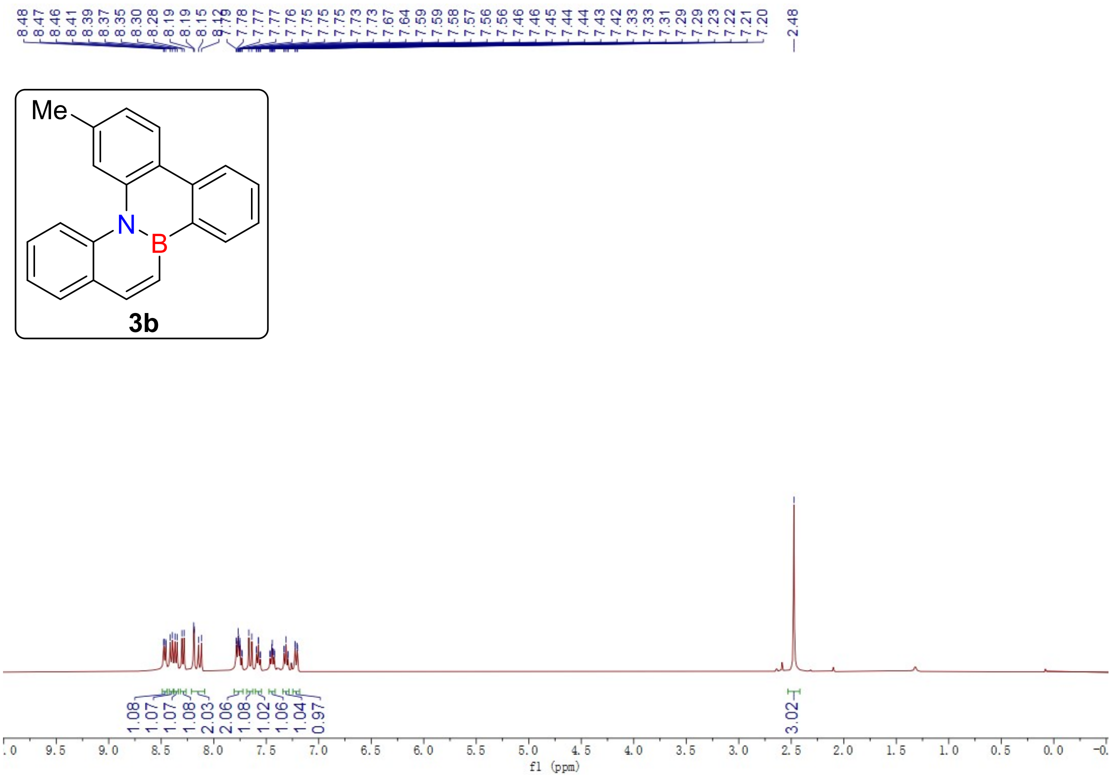

14-methyldibenzo[c,e]benzo[5,6][1,2]azaborinino[1,2-a][1,2]azaborinine (3b)

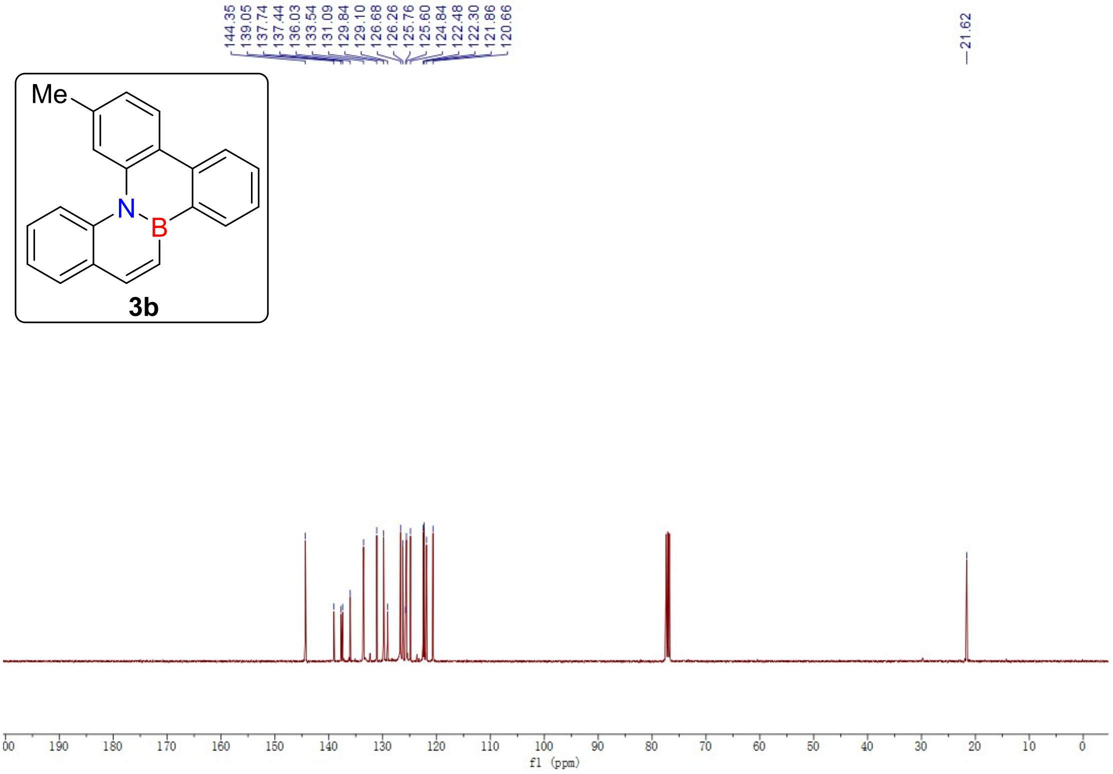

dibenzo[c,e]benzo[5,6][1,2]azaborinino[1,2-a][1,2]azaborinine-15-carbaldehyde (3c)

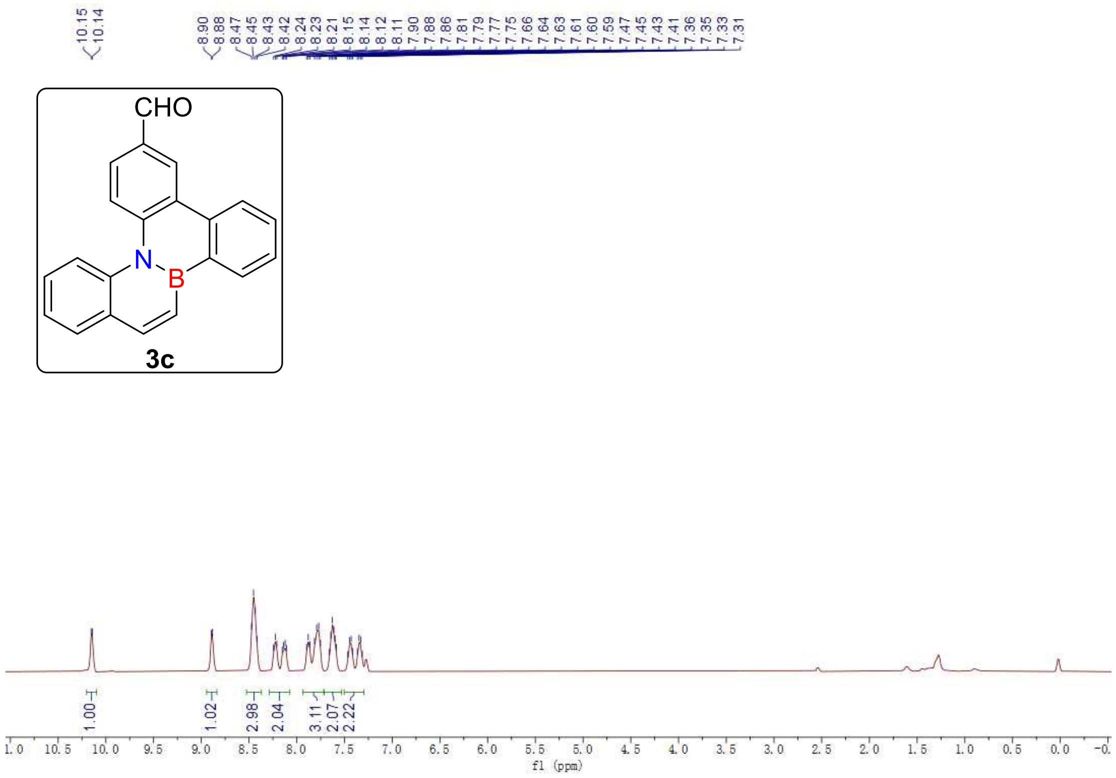

dibenzo[c,e]benzo[5,6][1,2]azaborinino[1,2-a][1,2]azaborinine-15-carbaldehyde (3c)

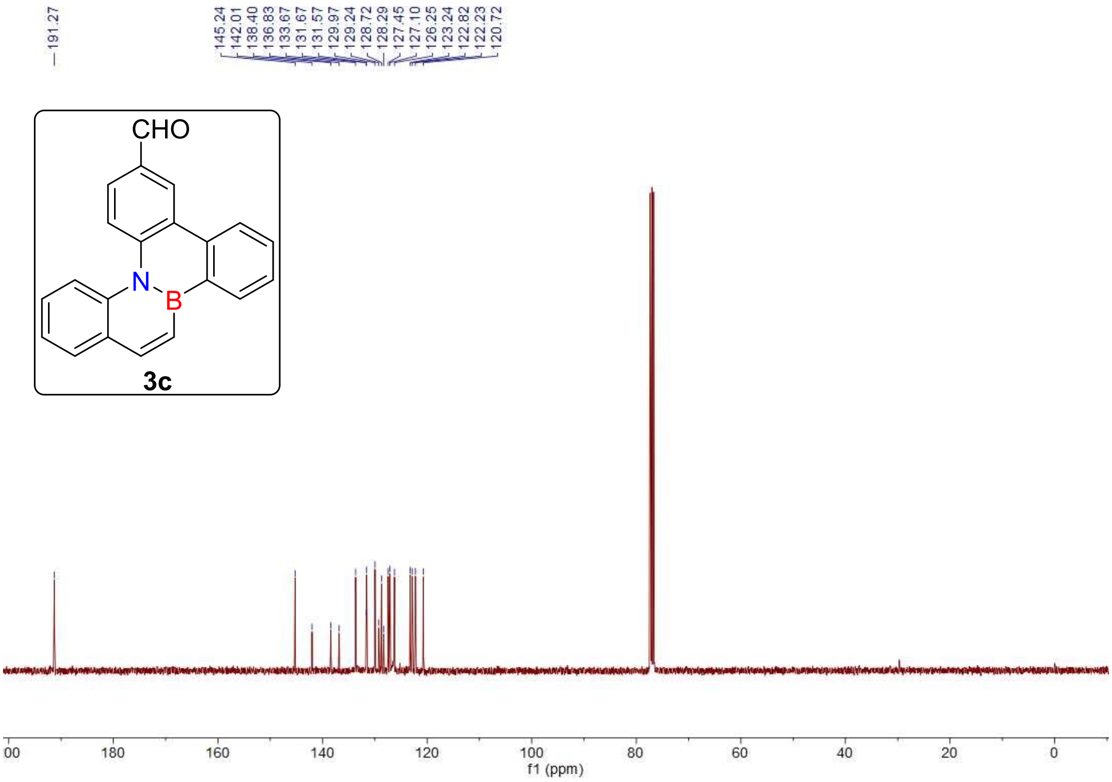

benzo[c]benzo[5,6][1,2]azaborinino[1,2-a]thieno[3,2-e][1,2]azaborinine (3d)

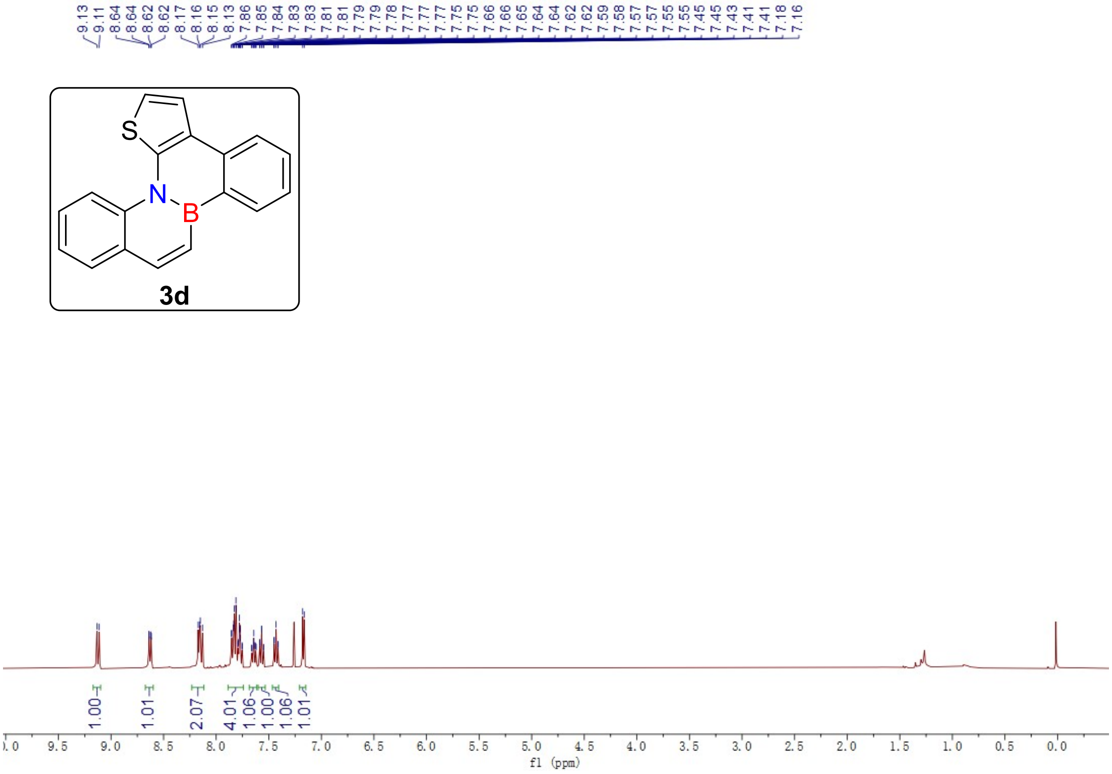

benzo[c]benzo[5,6][1,2]azaborinino[1,2-a]thieno[3,2-e][1,2]azaborinine (3d)

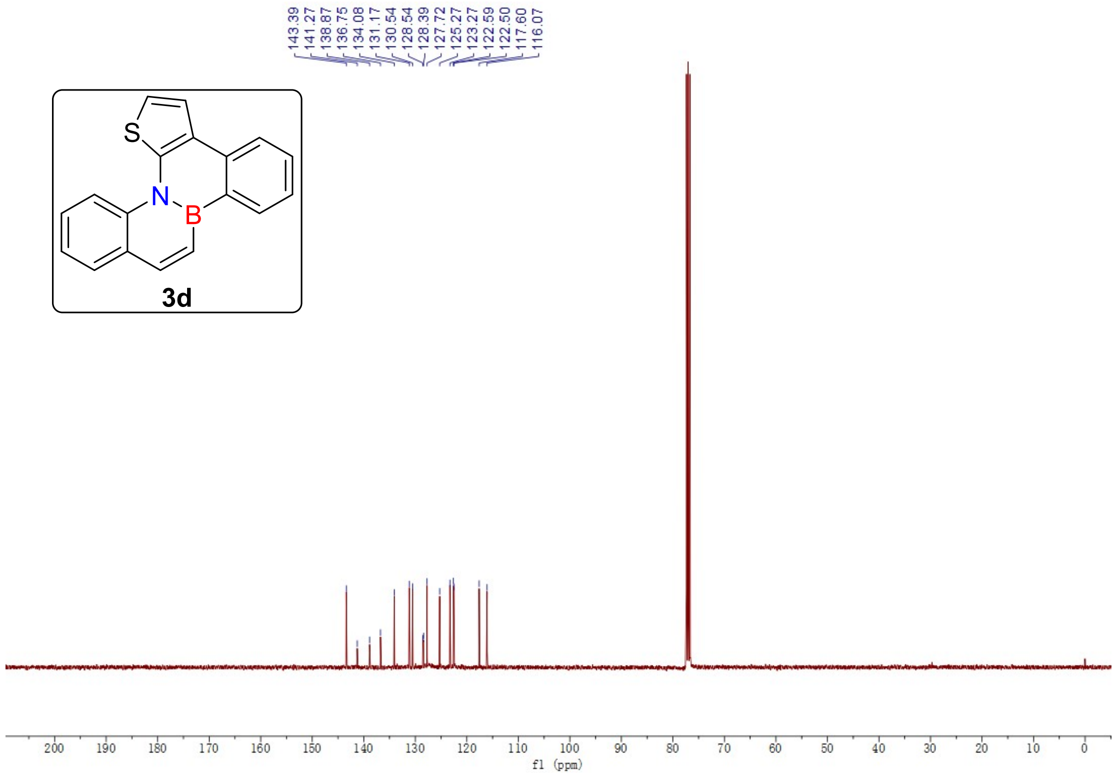

2-fluorodibenzo[c,e]benzo[5,6][1,2]azaborinino[1,2-a][1,2]azaborinine (3e)

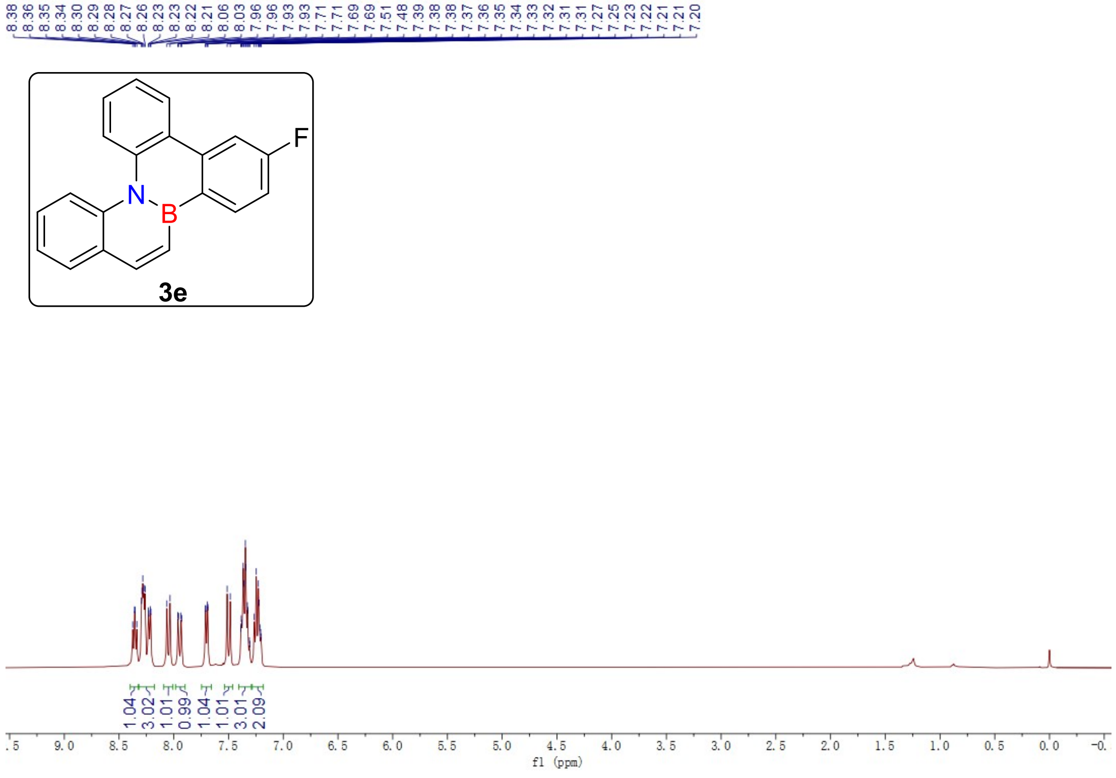

2-fluorodibenzo[c,e]benzo[5,6][1,2]azaborinino[1,2-a][1,2]azaborinine (3e)

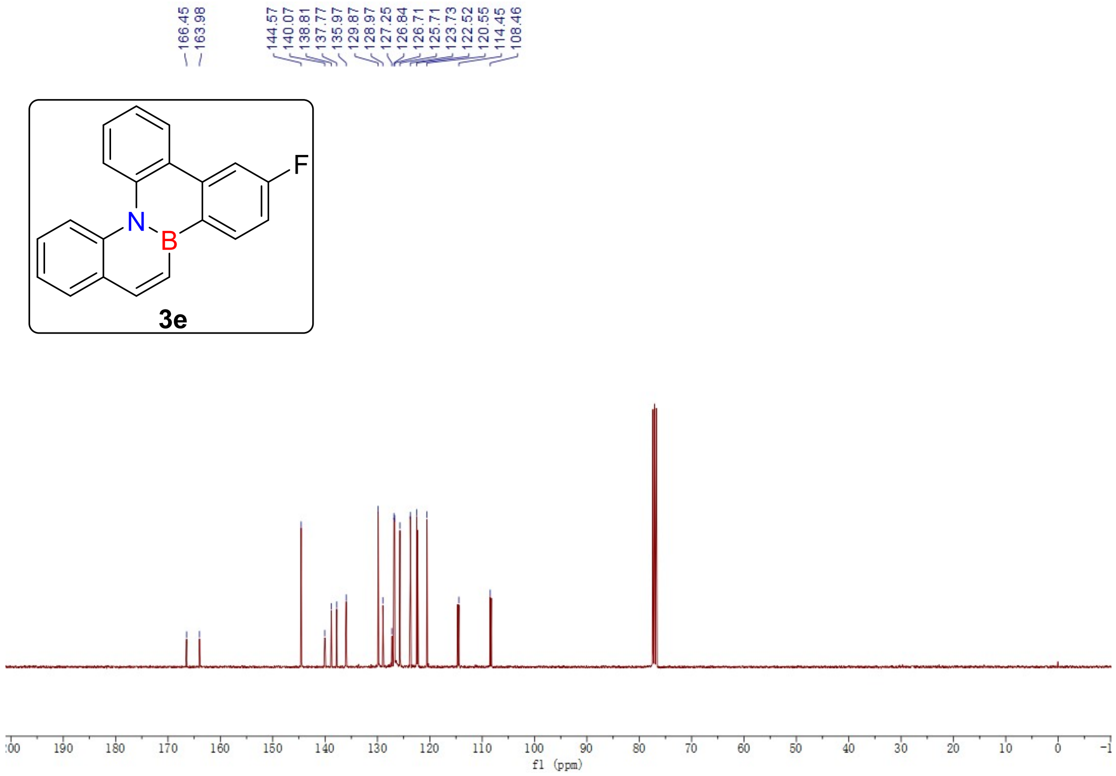

2,9-difluorodibenzo[c,e]benzo[5,6][1,2]azaborinino[1,2-a][1,2]azaborinine (3f)

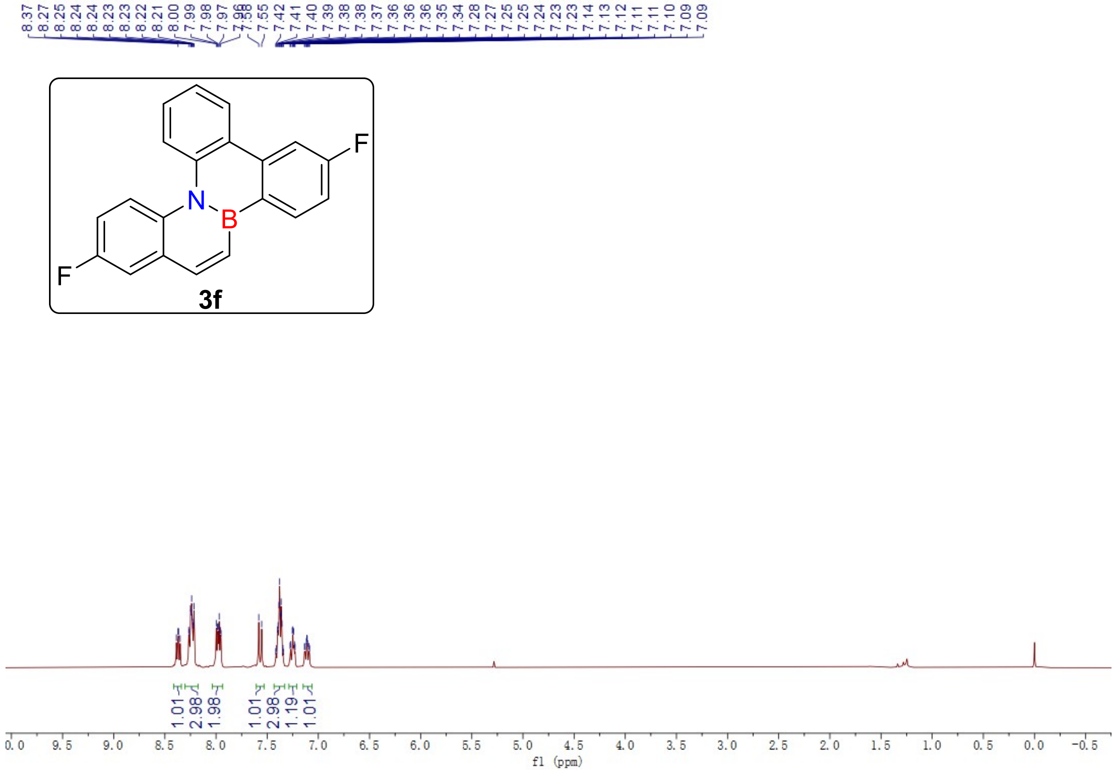

2,9-difluorodibenzo[c,e]benzo[5,6][1,2]azaborinino[1,2-a][1,2]azaborinine (3f)

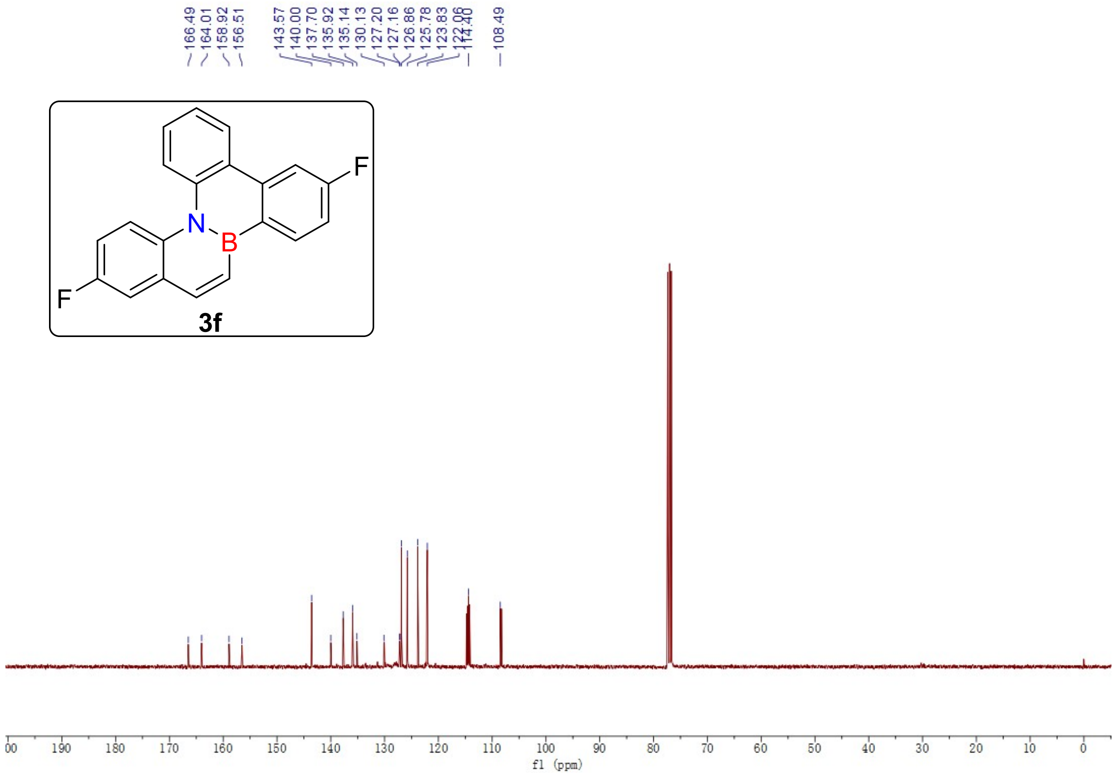

10H-benzo[e]dibenzo[3,4:5,6]borinino[1,2-b][1,2]azaborinine (4a)

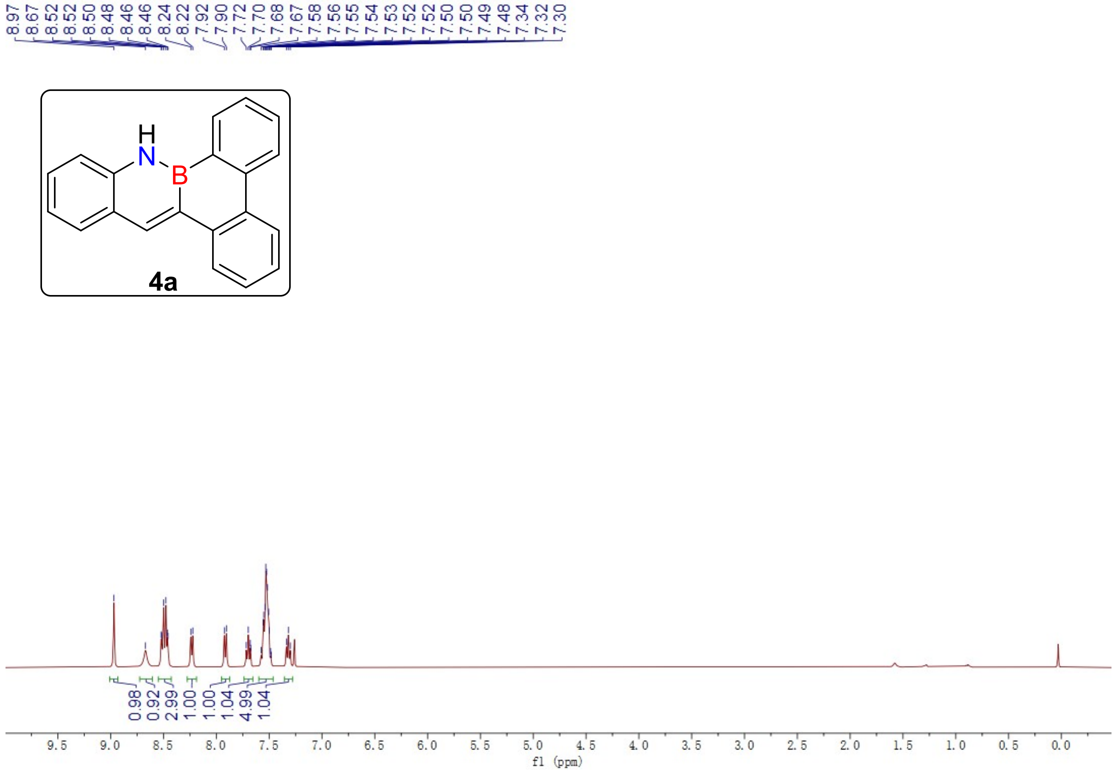

10H-benzo[e]dibenzo[3,4:5,6]borinino[1,2-b][1,2]azaborinine (4a)

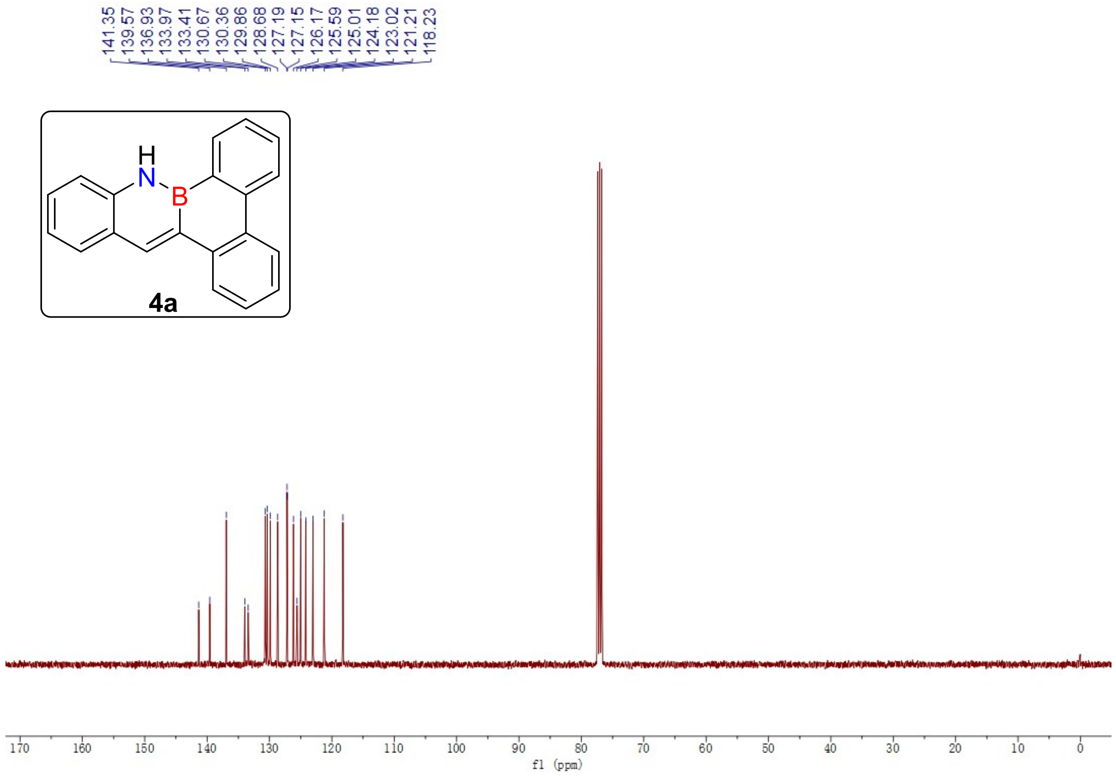

9H-benzo[e]benzo[5,6]thieno[3',4':3,4]borinino[1,2-b][1,2]azaborinine (4b)

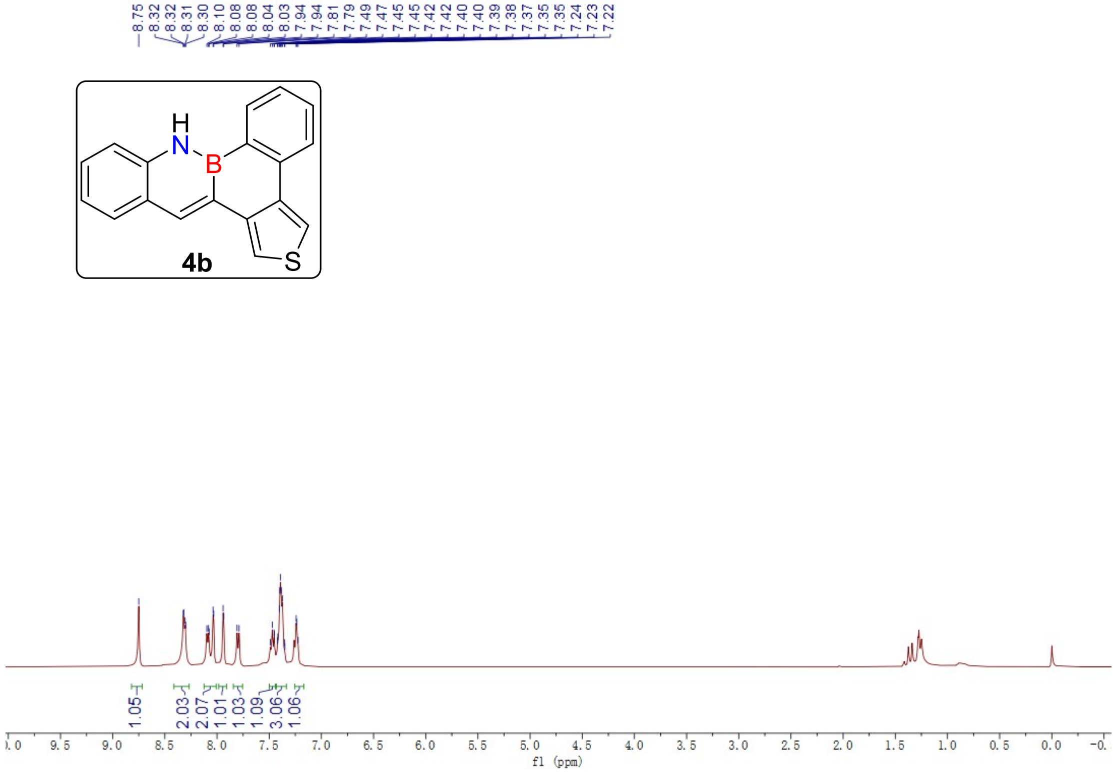

9H-benzo[e]benzo[5,6]thieno[3',4':3,4]borinino[1,2-b][1,2]azaborinine (4b)

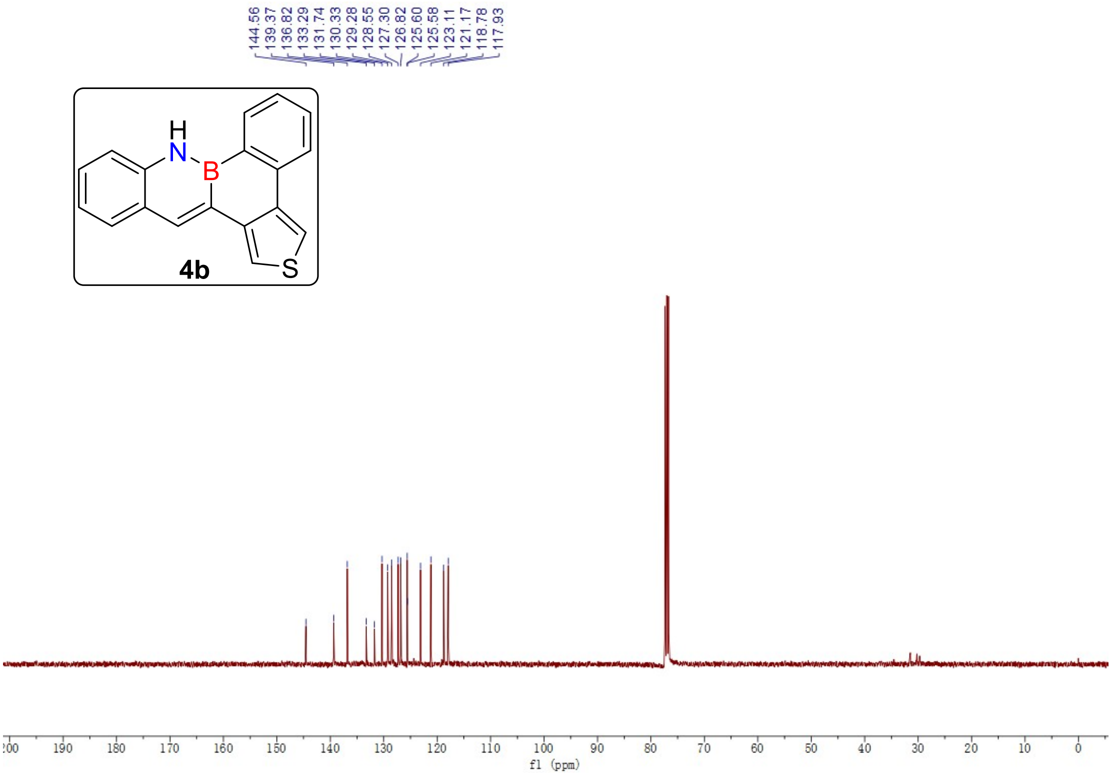

13-fluoro-10H-benzo[e]dibenzo[3,4:5,6]borinino[1,2-b][1,2]azaborinine (4c)

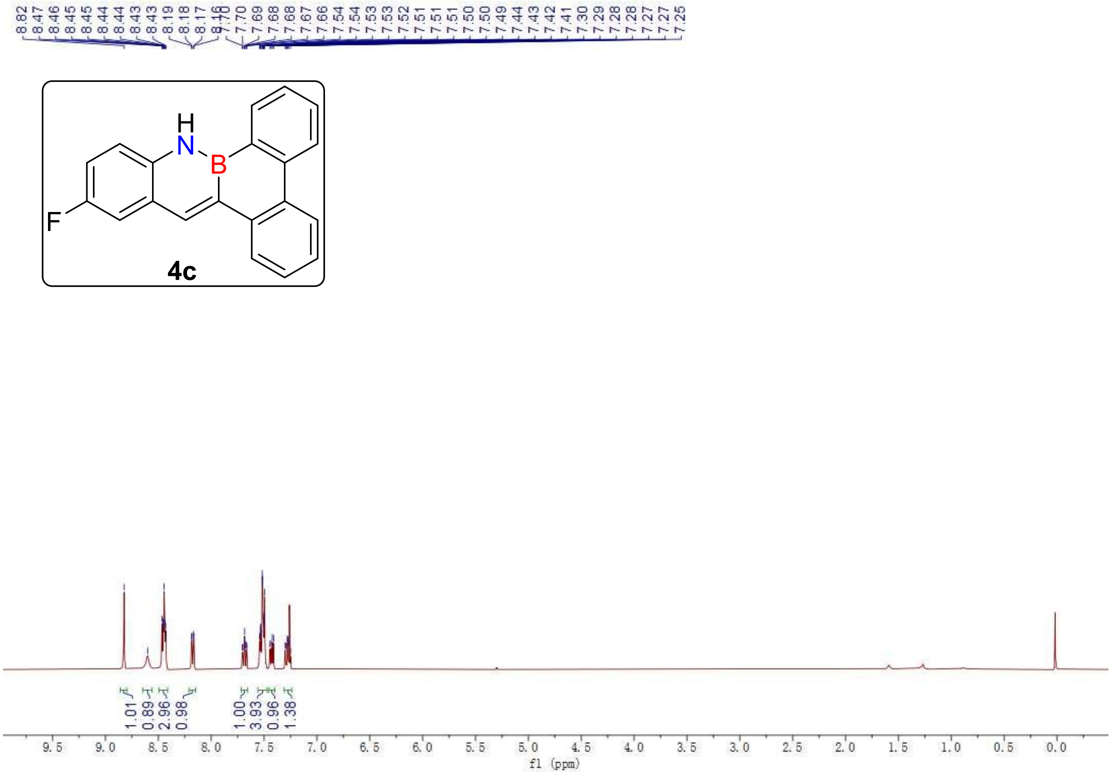

13-fluoro-10H-benzo[e]dibenzo[3,4:5,6]borinino[1,2-b][1,2]azaborinine (4c)

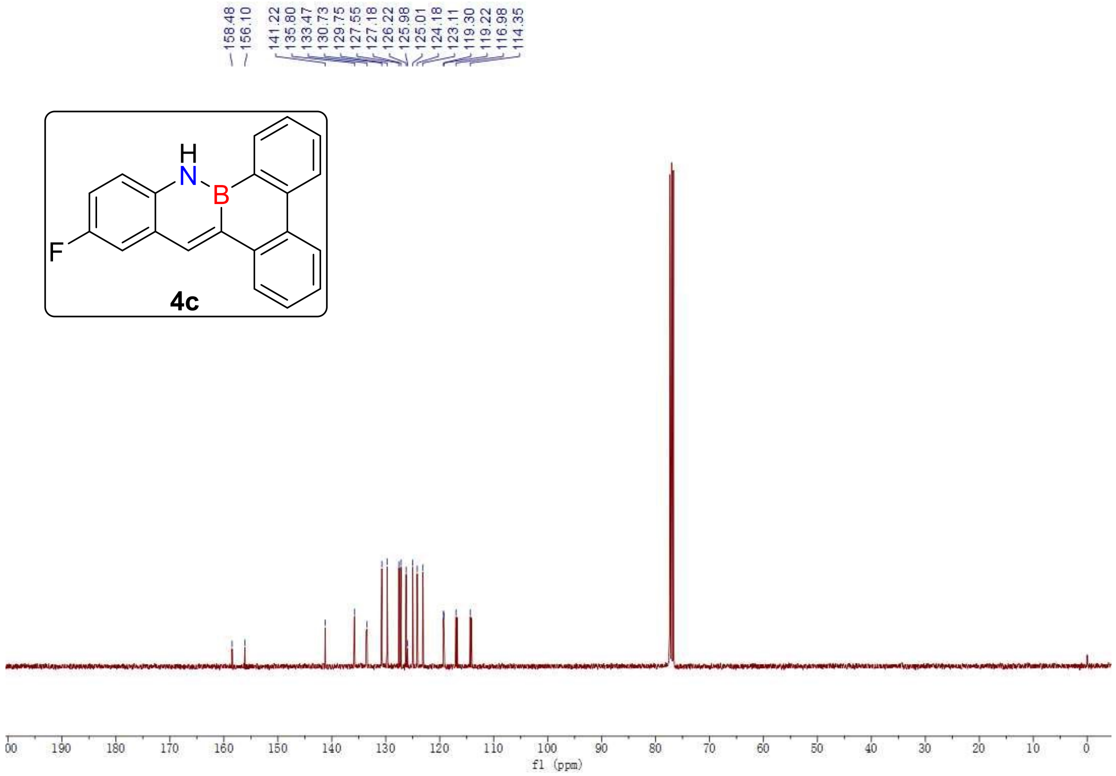
158.48
156.10
141.22
135.80
133.47
130.73
129.75
127.55
127.18
126.22
125.98
125.01
124.18
123.11
119.30
119.22
116.98
114.35
H
N
B
F
4c
00 190 180 170 160 150 140 130 120 110 100 90 80 70 60 50 40 30 20 10 0
f1 (ppm)
